# A boosting method for maximizing the partial area under the ROC curve

**DOI:** 10.1186/1471-2105-11-314

**Published:** 2010-06-10

**Authors:** Osamu Komori, Shinto Eguchi

**Affiliations:** 1 Prediction and Knowledge Discovery Research Center, The Institute of Statistical Mathematics, Midori-cho, Tachikawa, Tokyo 190-8562, Japan; 2 The Institute of Statistical Mathematics and Department of Statistical Science, The Graduate University for Advanced Studies Midori-cho, Tachikawa, Tokyo 190-8562, Japan

## Abstract

**Background:**

The receiver operating characteristic (ROC) curve is a fundamental tool to assess the discriminant performance for not only a single marker but also a score function combining multiple markers. The area under the ROC curve (AUC) for a score function measures the intrinsic ability for the score function to discriminate between the controls and cases. Recently, the partial AUC (pAUC) has been paid more attention than the AUC, because a suitable range of the false positive rate can be focused according to various clinical situations. However, existing pAUC-based methods only handle a few markers and do not take nonlinear combination of markers into consideration.

**Results:**

We have developed a new statistical method that focuses on the pAUC based on a boosting technique. The markers are combined componentially for maximizing the pAUC in the boosting algorithm using natural cubic splines or decision stumps (single-level decision trees), according to the values of markers (continuous or discrete). We show that the resulting score plots are useful for understanding how each marker is associated with the outcome variable. We compare the performance of the proposed boosting method with those of other existing methods, and demonstrate the utility using real data sets. As a result, we have much better discrimination performances in the sense of the pAUC in both simulation studies and real data analysis.

**Conclusions:**

The proposed method addresses how to combine the markers after a pAUC-based filtering procedure in high dimensional setting. Hence, it provides a consistent way of analyzing data based on the pAUC from maker selection to marker combination for discrimination problems. The method can capture not only linear but also nonlinear association between the outcome variable and the markers, about which the nonlinearity is known to be necessary in general for the maximization of the pAUC. The method also puts importance on the accuracy of classification performance as well as interpretability of the association, by offering simple and smooth resultant score plots for each marker.

## Background

The receiver operating characteristic (ROC) curve has been widely used in various scientific fields, in situations where the evaluation of discrimination performance is of great concern for the researchers. The area under the ROC curve (AUC) is the most popular metric because it has a simple probabilistic interpretation [1] and consists of two important rates used to assess classification performance: the true positive rate (TPR) and the false positive rate (FPR). The former is a probability of an affected subject being correctly judged as positive; the latter is that of an unaffected subject being improperly judged as positive. These two rates are shown to be more adequate to evaluate the classification accuracy than the odds ratio or relative risk [2]. However, the AUC has been severely criticized for inconsistency arising between statistical significance and the corresponding clinical significance when the usefulness of a new marker is evaluated [3]. Recently, Pencina et al. [4] propose a criterion termed integrated discriminant improvement and show the advantage over the AUC in the assessment of a new marker. In this context, the partial AUC (pAUC) is paid more attention than the AUC, especially in clinical settings where a low FPR or a high TPR is required [5-7].

Dodd and Pepe [8] propose a regression modeling framework based on the pAUC, and apply this framework to investigation of a relationship between a test result and the patient characteristics. Cai and Dodd [9] make some modifications to improve the efficiency of the estimation for parameters, and provide graphical tools for the model checking. In regard to classification problems, Pepe and Thompson [10] propose a method for deriving a linear combination of two markers that optimizes the AUC as well as the pAUC. However, as recognized by Pepe et al. [11], more general approaches are required when the number of markers is large. Moreover, the nonlinear combination of markers is necessary to maximize the AUC as well as the pAUC even in a simple setting such that normality is assumed to the distribution of markers [12]. However, the existing methods [10,13,14] only deal with the *linear *combination of markers.

In this paper, we propose a new statistical method designed to maximize the pAUC, as an extension of AUCBoost [12], using a boosting technique and the approximate pAUC. The approximation-based method makes it possible to *nonlinearly *combine more than two markers, based on basis functions of natural cubic splines as well as decision stumps. The resultant score plots for each marker enable us to observe how the markers are associated with the outcome variable in a visually apparent way. Hence, our boosting method attaches importance not only to the classification performance but also to the interpretation of the results, which is essential in clinical and medical fields.

This paper is organized as follows. In the Methods section, we present a new boosting method for the maximization of the pAUC after giving a brief review of the pAUC and the approximate pAUC. Then, we show a relationship between the pAUC and the approximate pAUC in Theorem 1, which justifies the use of the approximate pAUC in the boosting algorithm. In the Results and Discussion section, we compare the proposed method with other existing ones such as SDF [10], AdaBoost [15], LogitBoost [16] and GAMBoost [17]. In addition, we demonstrate the utility of the proposed method using real data sets; one of them is breast cancer data, in which we use both clinical and genomic data. In the last section, we summarize and make concluding remarks.

## Methods

### pAUC and approximate pAUC

#### Partial area under the ROC curve

Let *y *denote a class label for cases (*y *= 1) and controls (*y *= 0), and ***x ***be a vector of *p *markers as ***x ***= (*x*_1_, *x*_2_, ..., *x*_*p*_). Given a score function *F *(***x***) and a threshold *c*, we judge the subject as positive if *F *(***x***) ≥ *c*, and as negative if *F*(***x***) <*c*. The corresponding false positive rate (FPR) and true positive rate (TPR) are given as

where H is the Heaviside function: H(*z*) = 1 if *z *≥ 0 and 0 otherwise, and *g*_0_(***x***) and *g*_1_(***x***) are probability density functions given class 0 and class 1, respectively. Note that FPR and TPR are also dependent on the score function *F*. However, for the sake of simplicity, we abbreviate it when the abbreviation does not cause ambiguity.

Then, the ROC curve is defined as a plot of TPR against FPR when the threshold *c *moves on a real number line:

and the area under the ROC curve (AUC) is given as

In this setting, we consider a part of the AUC by limiting the value of FPR between *α*_1 _and *α*_2_, with corresponding thresholds *c*_1 _and *c*_2_, respectively:(1)

where 0 ≤ *α*_1 _<*α*_2 _≤ 1 (*c*_2 _<*c*_1_). In this paper, we set the values to be 0 and 0.1, respectively. However, it is also worth considering to take *α*_1_*>*0 and choose *α*_2_- *α*_1 _to be small enough, so that we essentially maximize TPR for the fixed range of FPR. Then, the pAUC can be divided into a fan-shaped part and a rectangular part:

Its probabilistic interpretation is offered by Dodd [18] and Pepe [19] as

Given samples from class 0  and class 1, the empirical form is expressed as(2)

where  and  are empirical values that are the closest to *α*_1 _and *α*_1 _respectively;  where  and  are thresholds determined by  and .

#### Approximate pAUC

As seen in Equation (2), the empirical pAUC is non-differentiable. Eguchi and Copas [20] use the standard normal distribution function to approximate the AUC, and applies an algorithms in order to maximize the AUC. Ma and Huang [13] and Wang et al. [14] employ the similar approximation to the AUC by a sigmoid function for multiple marker combination. Since there is no essential difference between the two approximations, we use the standard normal distribution for the approximation of the pAUC:

where *α*_1 _and *α*_2 _are defined in Equation (1), and H_*σ *_(*z*) is an approximation of H(*z*) by the standard normal distribution function, that is, H_*σ *_(*z*) = Φ(*z*/*σ*). Similarly, the corresponding empirical pAUC is defined as

where  and . A smaller scale parameter *σ *implies a better approximation of H(*z*).

### pAUCBoost with natural cubic splines

#### Boosting

Boosting is one of the most popular method for classification in machine learning community. The main concept is that the score function *F *is constructed based on various simple functions, termed weak classifiers. There exist many boosting methods according to the objective functions [15-17,21,22]. The seminal and important one is AdaBoost, whose objective function is the exponential loss and its algorithm with the iteration number *T *is as follows.

1. Start with a score function *F*_0_(*x*_*i*_) = 0, *i *= 1, 2, ..., *n*, where *n *= *n*_0 _+ *n*_1_.

2. For *t *= 1, ..., *T*

(a) Calculate the weights *w*_*t*_(*i*)

(b)For , find the best weak classifier *f*_*t*_(3)

where ℱ*Ada *is a set of weak classifiers taking values 1 or -1, and I(·) is the indicator function.

(c) Calculate the coefficient *β*_*t*_(4)

(d) Update the score function as

3. Finally, output a final score function as

Based on this iterative procedure, we propose the pAUCBoost algorithm after defining the object function.

#### Objective function

We construct a score function *F*(***x***) in an additive model for the maximization of the pAUC:(5)

where *F*_*k*_(*x*_*k*_) is the *k*-th component of *F*(***x***), and the plot of *F*_*k*_(*x*_*k*_) against *x*_*k *_is called a score plot that describes the association between *x*_*k *_and an outcome variable. The subset of weak classifiers for *x*_*k *_is given as

where *N*_*k, l *_(*x*_*k*_) is a basis function of *x*_*k *_for representing a natural cubic spline with *m*_*k *_knots, and *Z*_*k, l *_is a standardization factor that makes the heights of *N*_*k, l*_'s uniform. Thus, *F*_*k *_(*x*_*k*_) in Equation (5) has the following expression.

where *β*_*l*_'s are coefficients that are calculated in the pAUCBoost algorithm. Then, the set of weak classifiers that we use in pAUCBoost is defined as

In this setting, the objective function we propose is given as(6)

where  is the second derivative of *F*_*k*_(*x*_*k*_) and *λ *is a smoothing parameter that controls the smoothness of *F*(*x*). It is rewritten as(7)

Therefore, we have(8)

We remark that the scale parameter *σ *in the definition of  in Equation (6) can be fixed to 1 because of Equation (8). Hence, we redefine the objective function as

without loss of generality.

The maximum value that is attained by a set of (*F*_1_, *F*_2_, ..., *F*_*p*_) can take the larger value by replacing the functions with *p *sets of natural cubic splines. This can be proved in the same way as the result of generalized additive models [23], because the penalty term is the same. Hence, we find that the maximizer of the pAUCBoost objective function is the natural cubic spline.

#### pAUCBoost algorithm

Using weak classifiers *f *'s ∈ ℱ, we construct a score function *F *for the maximization of the pAUC. Note that the coefficient *β *cannot be determined independently of the weak classifier, so we denote it as *β*(*f*) in the following algorithm.

1.Start with a score function *F*_0_(***x***) = 0 and set every coefficient *β*_0_(*f*) to be 1 or -1, so that the candidates of the initial score function have positive or negative derivatives.

2. For *t *= 1, ..., *T*

(a) For all *f *'s ∈ ℱ, calculate the values of thresholds  and  of *F*_*t-1 *_+ *β*_*t-1 *_(*f*)*f*.

(b) Update *β*_*t*-1_(*f*) to *β*_*t*_(*f*) with a one-step Newton-Raphson iteration.

(c) Find the best weak classifier *f*_*t*_(9)

(d) Update the score function as(10)

3. Finally, output a final score function as

The dependency of the  on thresholds  and  makes it necessary to pick up the best pair *(β_*t*_(f_*t*_*), *f*_*t*_) at the same time in step 2.(c). This process is quite different from that of AdaBoost, in which *β*_*t *_and *f*_*t *_are determined independently in Equations (3) and (4). Because of the dependency and the difficulty of getting the exact solution of *β*_*t*_(*f*_*t*_), the one-step Newton-Raphson calculation is conducted in the boosting process. The one-step Newton-Raphson update is also employed in LogitBoost [16] and GAMBoost [17]. The details of the pAUCBoost algorithm are given in additional file 1: Details of the pAUCBoost algorithm.

#### Tuning procedure

We conduct *K*-fold cross validation to determine the smoothing parameter *λ *and the iteration number *T*. We divide the whole data into *K *subsets, and calculate the following objective function.

where *F*^(-*i*) ^denotes a score function that is generated by the data without *i*-th subset, and  is  calculated by the *i*-th subset only. The optimal parameters are obtained at the maximum value of pAUC_cv_(*λ*, *T*) in a set of grid points (*λ*, *T*). In the case where the values of the pAUC_cv_(*λ*, *T*) are unstable, we calculate the pAUC_cv_(*λ*, *T*) 10 times and take the average to determine the optimal parameters. In our subsequent discussion, we set *K *= 10 and explicitly demonstrate the procedure in the section regarding real data analysis.

### Relationship between pAUC and approximate pAUC

We investigate the relationship between the pAUC and the approximate pAUC, which gives a theoretical justification of the use of the approximate pAUC in the pAUCBoost algorithm.

**Theorem 1**. *For a pair of fixed α_1 _and α_2_, let*

where *γ *is a scalar, Λ(***x***) = *g*_1 _(***x***)/*g*_0_(***x***) and *m *is a strictly increasing function. Then, Ψ(*γ*) is a strictly increasing function of *γ*, and we have

See additional file 2: Proof of Theorem 1 and Corollary 1 for the details. Note that Theorem 1 holds for the approximate pAUC by a sigmoid function, so it also gives the justification of the AUC-based methods of Ma and Huang [13] and Wang et al. [14], as a special case where *α*_1_* = 0 *and *α*_2 _= 1. As proved in Eguchi and Copas [20] and Mcintosh and Pepe [24], the likelihood ratio Λ(***x***) is the optimal score function that maximizes the AUC as well as the pAUC. In general, the Bayes risk consistency has been well discussed under an assumption of convexity for a variety of loss functions [25]. Theorem 1 suggests a weak version of the Bayes risk consistency for the nonconvex function in the limiting sense.

We also have a following corollary from Theorem 1.

**Corollary 1**. *For any score function F, let*

*where η is a score function, and γ is a scalar. For a fixed FPR of F*_*γη*_*, the TPR of F*_*γη*_*becomes a increasing function of *γ *if and only if *η * = m*(Λ)*, where m is a strictly increasing function*.

See additional file 2: Proof of Theorem 1 and Corollary 1 for the details. Note that the corollary holds for any FPR in the range of (0,1). Hence, we find that the score function that moves every and all TPR's upward from the original positions, is nothing but the optimal score function derived from likelihood. This fact is not derived from the Neyman-Pearson fundamental lemma [26], from which *m*(Λ) is proved to maximize the AUC as well as pAUC. This corollary characterizes another property of the optimal score function *m *Λ.

## Results and Discussion

### Simulation studies

**Figure 1:**
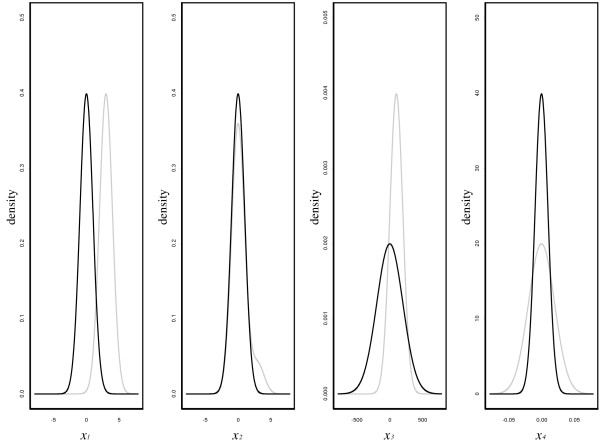
**Illustration of simulation setting**. Illustration of four different types of sample distributions for class 0 (black) and class 1 (gray).

We compare the performance of pAUCBoost with that of the smooth distribution-free (SDF) method proposed by [10] in a two-dimensional setting, and with those of other existing boosting methods: AdaBoost, LogitBoost and GAMBoost in a higher-dimensional setting. The simulation setting is similar to that of [27]. Suppose that there are four types of sample distributions for each class, *y *= 0 or *y *= 1, as shown in Figure 1. The first panel shows an ideal situation, where we see very little overlap between the two class-conditional distributions. The second situation is of practical interest for disease screening, where FPR must be restricted to be as small as possible, in a case where invasive or costly diagnostic treatments will follow. A small portion of samples from class 1 (cases) is clearly distinguishable from the bulk of samples from class 0 (controls). On the other hand, in the third situation, cases are completely within the range of controls, and therefore not useful for disease screening. The fourth situation is similar to the second one, but some of the samples from cases deviate from controls clearly on both side of the distribution, rather than only on one side. This situation could be worth consideration in a case where high TPR is required with very low FPR in the same way as in the second situation.

In the simulation study, we apply pAUCBoost with  = 0 and  = 0.1. The training data set contains 50 controls and 50 cases, and the accuracy of the performance is evaluated based on 100 repetitions using test data sets of size 1000 (500 for each class).

#### Comparison with SDF

**Figure 2:**
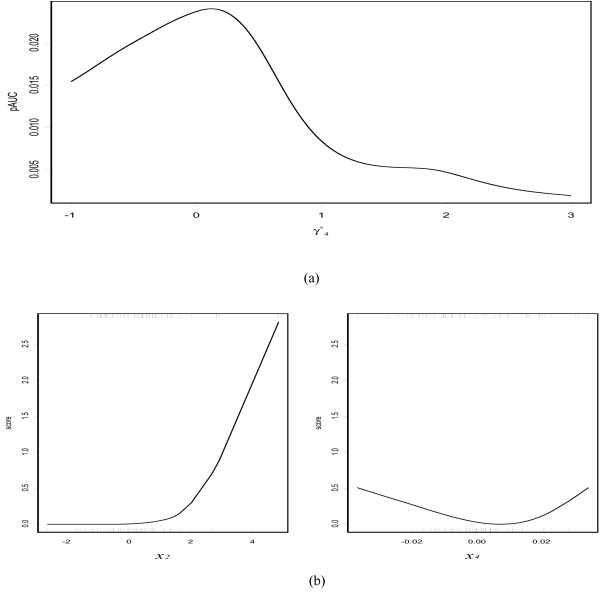
**Comparison of SDF method and pAUCBoost method**. (a) Illustration of the estimated value of pAUC by SDF method, where  and 2-1/*γ*_4 _otherwise; (b) the resultant score plots by pAUCBoost. The rug plot along the bottom of each graph describes the observations from class 0; the rug plot along the top of each graph describes those from class 0.

We consider the second situation, where we assume normality distributions such as  and  with mixing proportion *π *= 0.9, and the last situation: . That is, the conditional probability density function of a class label *y *given ***x ***is given by(11)

where Λ_1_(***x***) is the likelihood ratio:(12)

and *ϕ*(*z*) is the standard normal density function. The resultant mean value (and the 95 percent confidence interval) of the pAUC based on pAUCBoost turns out to be 0.017 (0.012, 0.020), and the value of SDF to be 0.011 (0.005, 0.017). This large difference is because SDF assumes linearity of the score function of *F*(***x***) as

and the coefficient of *x*_4 _is estimated by SDF to be around 0 as shown in Figure 2 (a), under the condition that *λ*_2 _is fixed to 1. On the other hand, pAUCBoost considers the nonlinearity of *F*(***x***) as

as shown in Figure 2(b). The left panel shows the score plot of *x*_2_, and the right one shows that of *x*_4_. The pAUCBoost clearly captures the nonlinearity of *F*_4 _(*x*_4_), where one of the optimal score function in this setting is derived from Equation (12) as

Note that the ROC curve is invariant to a monotone transformation of the score function.

Although a nonlinear transformation could be applied to the data in advance, it is not practical to examine all marginal distributions and decide the appropriate transformations in general situations. Hence, it is better to take the nonlinearity into consideration in the method itself in this way.

We have also confirmed that the performance of pAUCBoost is compatible with that of SDF, in a setting when linearity of the score function is reasonable. We have averages of 0.013 (0.007, 0.017) and 0.013 (0.011, 0.015 pAUCBoost and the SDF method, respectively, under the situation that *x*_4 _is also distributed as  and . That is, the conditional probability density function of *y *given *x *is given same way as Equation (11):

where

The one of the optimal score function is given by

It is interesting to note that almost the same results are obtained by these quite different statistical methods. SDF uses the estimated values of pAUC to derive a score function; on the other hand, pAUCBoost directly uses the empirical value of the approximate pAUC in the algorithm.

#### Comparison with other boosting methods

We focus on only the most practical situation in disease screening such as the second situation in Figure 1. Pepe et al. [27] show the utility of the use of the pAUC, in selection of potential genes that are useful for discrimination between normal and cancer tissues. The point is that the value of pAUC reflects the overlap of two distributions of controls and cases, so that we can select genes that are suitable for the purpose of further investigation. For example, some overexpressed genes encourage us to investigate the corresponding protein products. However, the task of how to combine the selected genes for better discrimination is still pending.

Suppose we select 50 genes by a filtering procedure, which are closely correlated each other, such that 50-dimensional gene vectors for class 0 and class 1 are distributed as  and , respectively. The covariance matrices are designed as **Σ**_0 _= 0.95 × ***W***_0 _+ 0.05 × ***I ***and **Σ**_1 _= 0.95 × ***W***_1 _+ 0.05 × ***I***, where ***W***_0 _and ***W***_1 _are 50 × 50 matrices that are sampled from Wishart distribution with the identity matrix and 10 degrees of freedom at every repetition of the simulation. The identity matrix ***I ***is added for avoiding the singularity of the covariance matrices. These matrices are normalized to have 1's on the diagonal part in the similar way to the simulation setting of Zhao and Yu [28], and the range of the correlations turns out to be about between *0.8 *and *-0.8. *Then, we randomly replace 10 percent of samples from class 1 with those that are distributed as  for each gene, so that each gene is informative in the sense of the pAUC as shown in the second situation of Figure 1.

**Figure 3:**
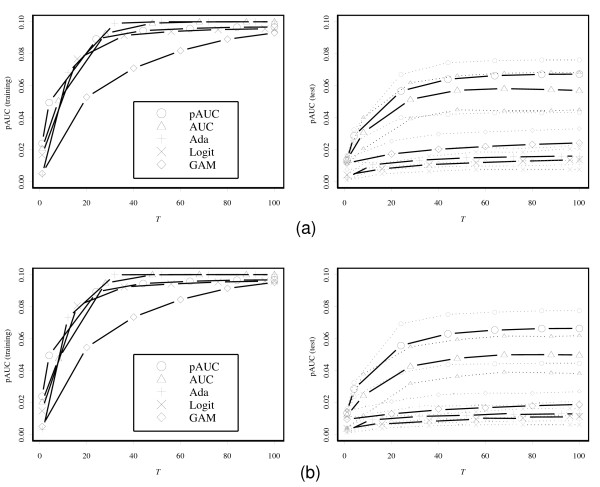
**Results of simulation study based on the values of the pAUC**. (a) The results of the pAUC with FPR between 0 and 0.1 for training data (left panel) and test data (right panel) with only informative genes. The gray dashed lines indicate the 95% confidence bands. (b) the results of the pAUC with noninformative genes added.

Figure 3(a) shows plots of the average of the pAUC against iteration number *T *for five boosting methods. For all the boosting methods, the values of the pAUC based on the training data almost reach the upper bound values 0.1 after a number of iterations. However, the values based on the test data show clear differences. The pAUCBoost properly detects the small difference of the two distributions illustrated in the second panel in Figure 1, and shows the best performance. On the other hand, AdaBoost, LogitBoost and GAMBoost cannot distinguish the two groups at all.

**Figure 4:**
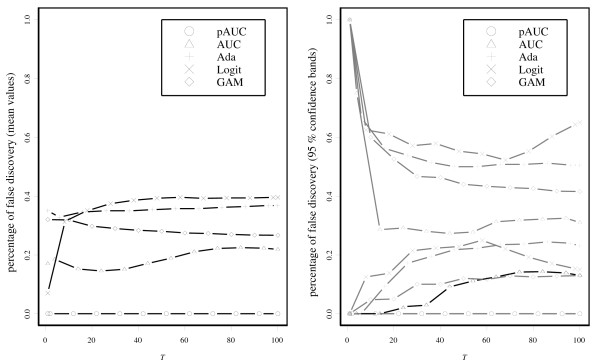
**Results of simulation study based on the marker selection**. The mean values of percentage of false discovery (left panel) and 95% confidence bands (right panel) for each boosting method. The horizontal axis denotes the iteration number of *T*. The lower sides of the 95% confidence bands of AUCBoost are shown by the heavy black line to emphasize the difference from those of pAUCBoost.

Next for illustration of the gene selection of pAUCBoost, we added some noninformative genes to the 50 genes above, i.e., genes that are assumed to be normally distributed with the same mean and the same covariance matrix: , where ∑ is generated in the same way as above. The results in the left panel of Figure 3(b) are the almost the same as those in Figure 3(a). However, we can find a clear difference between the right panels. The performances of all methods except for pAUCBoost go down on a relatively large scale. We can observe that the mean values of the pAUC by pAUCBoost are above the upper sides of the 95% confidence bands of those by AUCBoost after around *T *= 20. This is mainly because of "false discovery", or selection of noninformative genes by chance. Figure 4 shows the resistance of pAUCBoost to false discovery. The mean values of percentage of false discovery (the number of selected noninformative genes over the number of selected genes) are plotted in the left panel; the 95 percent confidence bands (gray lines) are plotted in the right panel, against the iteration number *T*, respectively. We see that the boosting methods other than pAUCBoost select noninformative genes from the early stage of boosting procedure. The difference of performance of pAUCBoost from the others is 95% significant after around *T *= 15 as shown in the right panel. The upper side bands of the 95% confidence bands reached 1 at the very beginning of the iteration for AUCBoost, AdaBoost, LogitBoost and GAMBoost. The boosting methods other than pAUCBoost clearly suffer from false discovery. pAUCBoost seems to have an advantage because it focuses on the essential part of the sample distribution in the sense of the pAUC.

Mainly, there are two types of weak classifiers: smoothing splines and decision stumps. Buhlmann and Yu [21] proposed to use smoothing splines in the *L*_2 _Boost algorithm, and Tutz and Binder [17] used B-splines in GAMBoost. However, the way of fitting the weak classifiers in pAUCBoost is different from those methods. Our algorithm updates a score function with a basis function of a natural cubic spline for one marker in Equations (9) and (10). On the other hand, their algorithms update a score function with *a set *of basis functions for one marker. Hence, our resultant score functions have tendency to have simpler forms (See the illustrations of score plots in the next section), which also leads to simple interpretation of the association between the markers and the outcome variable. Note that there exists a trade-off between the simplicity and the number of markers necessary for good performance of discrimination. However, the simplicity depends on the number of basis functions used for the selected markers, so the more complicated association can be expressed by increasing the number of the basis functions.

In AdaBoost and LogitBoost, decision stumps are used as weak classifiers [29,30] . The advantage of using decision stumps is that we can apply the boosting methods independently of the scale of the marker values. Hence, the decision stump-based method is resistant to outliers, which often occur in real data. However, it easily suffers from false discovery, as clearly shown in simulation studies. This causes poor performance in a setting where non-informative genes are mixed with informative ones. We have also confirmed that pAUCBoost with decision stumps for weak classifiers shows worse performance than that of pAUCBoost with natural cubic splines. Hence, we have to be much careful about which weak classifiers to be employed. It depends on the types of markers or the purpose of the analysis we are engaged in.

## Application of pAUCBoost to real data

### Breast cancer data

The breast cancer data of van't Veer et al. [31] contains not only gene expression profiles but also clinical markers such as Age, age of patients; Size, diameter of breast cancer; Grade, tumour grade; Angi, existence or nonexistence of angioinvasion; ERp, ER expression; PR_p_, PR expression; and Lymp, existence or nonexistence of lymphocytic infiltrate. First, we apply AUCBoost to these clinical markers and investigate their utility. The weak classifiers we use are natural cubic splines for continuous markers (Age and Size), and decision stumps to discrete or categorical markers. Second, we apply pAUCBoost with  = 0 and  = 0.1 to the gene expression data after a pAUC-based filtering procedure proposed by Pepe et al. [27]. The training data set and the test data set are the same as those in [31], that is, 44 patients with good prognosis and 34 patients with distant metastases for training data, and 7 and 12 patients for test data, respectively.

**Figure 5:**
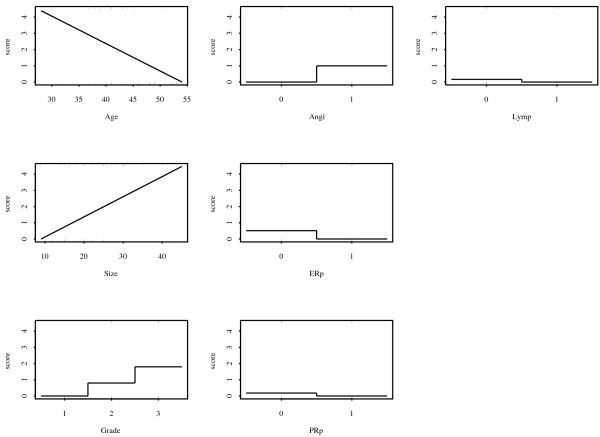
**Score plots of clinical markers in breast cancer data**. Score plots of clinical markers that describe the association between the markers and the outcome variable. The rug plot at the bottoms of each score plot shows the observations from patients with good prognosis; the rug plot for patients with distant metastases is described at the top of each score plot.

Figure 5 shows the results of the score plot generated by AUCBoost with *λ *= *0.01 *and *T *= 20, which were determined by a 10-fold cross validation. The Age and Size showed almost linear association with the prognosis, and a tendency to develop metastases increased as the value of Grade. The patients with negative ER and negative PR were estimated to have high risk of metastases, which are consistent with the result of van't Veer et al. [31]. The order of description of the score plots is in accordance with that of markers selected in the AUCBoost algorithm. Hence, Age has the largest contribution to the value of the AUC. The order is from the upper left panel to the lower right panel, so the second important marker is Size and the last one is Lymp. We have found that the values of the AUC for training and test data are 0.846 and 0.964, respectively. These results are comparable to those of van't Veer et al. [31] that were derived from the gene expression data: 0.882 and 0.869, respectively. This means that clinical markers themselves also have the ability to discriminate to some extent the patients with good prognosis from those with metastases.

**Figure 6:**
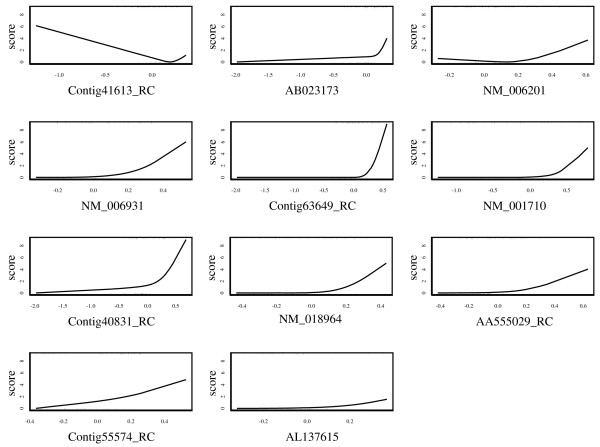
**Score plots of gene expressions in breast cancer data**. Score plots of the selected 11 genes that describe the association between the genes and the outcome variable. The rug plot at the bottoms of each score plot shows the observations from patients with good prognosis; the rug plot for patients with distant metastases is described at the top of each score plot.

**Figure 7:**
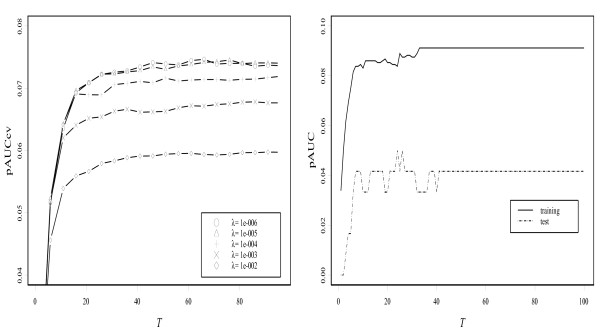
**Results of the pAUC**. The results of 10-fold cross validation with different values of smoothing parameter *λ *and iteration number *T *(left panel); the results of the values of pAUC for training data (solid) and test data (dashed) by pAUCBoost, as a function of *T *with *λ *= 10^-6 ^(right panel).

**Figure 8:**
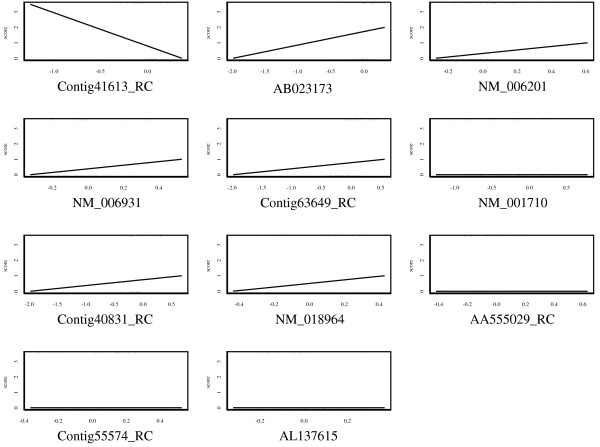
**Linear score plots of gene expressions in breast cancer data**. Score plots of the selected 11 genes generated by pAUCBoost using only linear weak classifiers.

**Figure 9:**
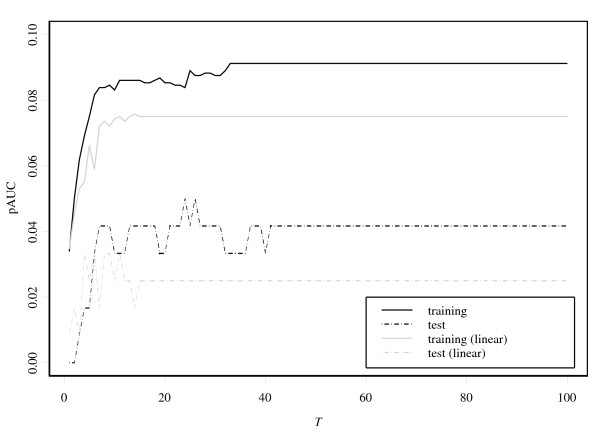
**Comparison between linear and nonlinear score functions**. Comparison based on the values of the pAUC between linear and nolinear score functions generated by pAUCBoost.

Next, we analyze the gene expression data as follows. The informative genes were selected, in the same way as [31], from the total of 25000 genes according to the criteria that the genes are two-fold regulated and that the p-values are less than 0.01 in more than 3 patients. Then, the approximately 5000 filtered genes were ordered based on their values of the pAUC with  = 0 and  = 0.1. In order to assess the variability of the top genes, we used the probability of gene selection proposed by Pepe et al. [27], that is(13)

where *k *was set to 100 in this analysis and this probability was calculated by 1000 bootstrap resampling replications. Table 1 shows the results of the top 30 genes ranked by *P*_*g *_(100), along with the values of pAUC and AUC calculated from the original data. We picked up significant genes with P_*g *_(100) > 0.5, and applied pAUCBoost to the 11 genes. The score plots in Figure 6 describe the nonlinear association between gene expressions and the prognosis. Among the 11 genes, Contig41613_RC showed a nonlinear and nonmonotonic association. That is, the gene expression of the patients with metastases had large variance as shown by the rug plot, compared with that of patients with good prognosis, which had a tendency to take small absolute values and concentrate around the origin. The nonlinearity of the associations can be captured by pAUCBoost in this way. The values of tuning parameter *λ *and *T *were determined to be 10^-6 ^and 65 by 10-cross validation, as described in the left panel in Figure 7. The value of *A *is very small, and it seems to be ignorable. However, since the value of *A *has an implicit role to control the accuracy of approximation of the pAUC as seen Equations (7) and (8), it should not be set to 0. The right panel in Figure 7 shows the pAUC for training (solid) and test (dashed) data, as a function of *T *with *λ *= 10^-6^. We saw that both of the values for training and test data are more than 3 times larger than those of van't Veer et al. [31]: 0.025 and 0.0084, respectively. Finally, we confirmed that the nonlinearity of score function *F *as shown in Figure 6 played an important role for the classification performance. See the score plots that are generated by only linear basis functions of natural cubic splines in Figure 8, and resultant values of the pAUC in Figure 9. Both the values of the pAUC based on training data and those on test data changed for the worse. The results of other boosting methods, and the results for less stringent bounds on the values of  are presented in additional file 3: Supplementary results of breast cancer data analysis.

**Table 1:** The top 30 genes ranked by the probability of gene selection, and the values of the pAUC and AUC.

No	gene name	*P*_*g*_(100)	pAUC	AUC
1	Contig41613_RC	0.728	0.036	0.666
2	NM_006931	0.728	0.035	0.678
3	Contig40831_RC	0.706	0.037	0.672
4	Contig55574_RC	0.639	0.035	0.654
5	AB023173	0.636	0.034	0.684
6	Contig63649_RC	0.626	0.034	0.749
7	NM_018964	0.586	0.034	0.660
8	AL137615	0.571	0.033	0.655
9	NM_006201	0.541	0.032	0.664
10	NM_001710	0.520	0.032	0.638
11	AA555029_RC	0.519	0.032	0.708
12	NM_020386	0.490	0.030	0.699
13	Contig7558_RC	0.488	0.032	0.659
14	Contig51464_RC	0.482	0.030	0.668
15	NM_014246	0.474	0.032	0.613
16	NM_007359	0.463	0.032	0.696
17	NM_006148	0.450	0.029	0.661
18	NM_004163	0.442	0.029	0.729
19	Contig37562_RC	0.423	0.031	0.630
20	Contig55377_RC	0.416	0.029	0.726
21	Contig47405_RC	0.404	0.029	0.718
22	NM_012261	0.393	0.029	0.721
23	NM_014400	0.379	0.028	0.681
24	Contig44409	0.368	0.029	0.692
25	AL080059	0.364	0.027	0.801
26	Contig60864 RC	0.358	0.029	0.637
27	NM_003748	0.353	0.025	0.793
28	AL080110	0.349	0.026	0.652
29	AL122101	0.343	0.028	0.708
30	NM_018120	0.336	0.026	0.671

### Ovarian cancer data

**Figure 10:**
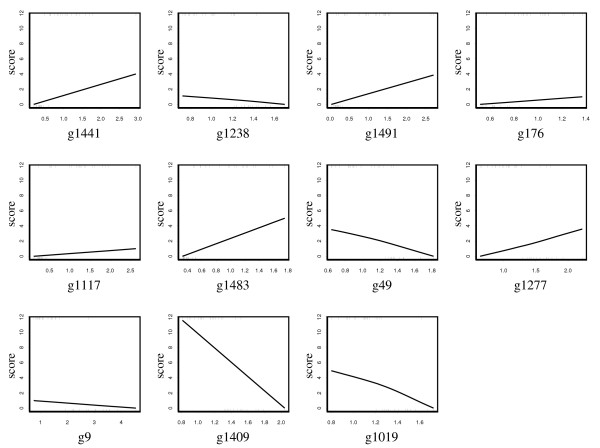
**Score plots of gene expressions in ovarian cancer data**. Score plots of the selected 11 genes by pAUCBoost based on ovarian cancer data. The rug plot at the bottoms of each score plot shows the observations from normal controls; the rug plot for ovarian cancer cases is described at the top of each score plot.

This dataset was analyzed by Pepe et al. [27] for illustration of their pAUC-based filtering procedure in Equation (13). It consists of 1536 genes spotted on the glass arrays, and is available from the website of a textbook by Pepe [19]. It includes 23 controls with normal ovarian tissues and 30 cases with ovarian cancers. We divided the whole data into training data and test data in the ratio of 2 to 1. That is, the first 15 controls and 20 cases in the original data are used for training data; the others are for test data. Using the training data only, we ranked the genes according to the value of the pAUC with  = 0 and  = 0.1, and assessed the variability as in the breast cancer analysis above. Then, we picked up 20 genes that satisfy *P*_*g*_(100) > 0.9 in the same way as Pepe et al. [27]. We found that there are 12 common genes to theirs, including g1483 that ranked best in their analysis. For these 20 filtered genes, we applied pAUCBoost and had the resultant score plots in Figure 10. As seen is Figure 10, the pAUCBoost selected 11 genes and attained the maximum value of the pAUC (0.1). Finally, we assessed the classification performance based on the independent test data, and had a high value of the pAUC (0.08). The classification is relatively easy, so the results of other boosting methods also reached the same values of the pAUC based on the independent test data.

### Leukemia data

**Figure 11:**
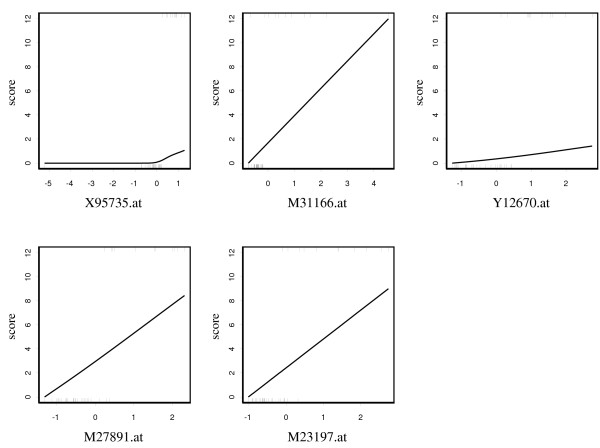
**Score plots of gene expressions in leukemia data**. Score plots of the selected 5 genes by pAUCBoost based on leukemia data. The rug plot at the bottoms of each score plot shows the observations from ALL; the rug plot for AML is described at the top of each score plot.

The third data we analyzed is leukemia data [32]. It contains 38 training samples and 34 test samples with 7129 genes for acute myeloid leukemia (AML) and acute lymphoblastic leukemia (ALL). We repeated the same procedure as the previous two analyses above using the 8 filtered genes that satisfy *P*_*g*_(100) > 0.9. We achieved the perfect classification performance regarding both training and test data sets, and had the score plots in Figure 11. The results of other boosting methods produced similar but a little worse values of the pAUC. That is, the values are more than 0.08 but less than 0.1 on the basis of test data.

## Conclusions

We have developed the pAUCBoost algorithm to maximize the pAUC based on the approximate pAUC in the additive model. The use of the approximate pAUC is justified by showing a relationship with the pAUC in Theorem

A resultant score function is decomposed componentwise into functions that are useful for understanding the associations between each marker and the outcome variable, as shown in real data analysis. Natural cubic splines that give the maximum of the pAUCBoost objective function are used for markers taking continuous values. In addition, using decision stumps for markers that take discrete or categorical values the proposed method enables us to treat various kinds of markers together.

We have also provided a consistent way to analyze gene expression data in the sense of the pAUC, as shown in the analysis of the breast cancer data, ovarian cancer data and leukemia data. The pAUC is shown to be useful by Pepe et al. [27] for selection of informative genes, some of which are overexpressed or underexpressed in cancer tissues. However, how to combine the selected genes and how to discriminate the cancer tissues from normal tissues, have not been addressed. We nonlinearly combined the genes ranked by the pAUC in order to produce a score function, by which the classification of controls and cases is done. Interestingly, we have found 4 genes in common with the 70 genes of van't Veer et al. [31]: Contig63649_RC, AA555029_RC, Contig40831_RC, NM_L006931. 6 genes among the selected 11 genes are related to protein coding. We also applied pAUCBoost to the 70 genes for comparison with the result from the 11 genes. We found that it yielded a poor result, especially about the value of pAUC for test data. Hence, pAUCBoost with FPR restricted to be small should be applied to the genes or markers that have gone through a pAUC-based filtering procedure beforehand. In the usual analysis setting, in which markers do not have especially high values of the pAUC, AUCBoost is preferable because of the stable performance due to the comprehensive information it can take into the algorithm.

## Authors' contributions

OK carried out the simulation study and the real data analysis. OK and SE are responsible for the algorithm of proposed method, the proof of Theorem 1 and Corollary 1 in Additional File 2. They drafted the manuscript and approved the final manuscript.

## Supplementary Material

Additional file 1 **Details of the pAUCBoost algorithm**. gives the details of the pAUCBoost algorithm. Click here for file

Additional file 2 **Proof of Theorem 1 and Corollary 1**. contains the details of the proof of Theorem 1 and Corollary 1. Click here for file

Additional file 3 **Supplementary results of breast cancer data analysis**. describes the supplementary results of breast cancer data, where the range of FPR is more relaxed. Click here for file
