# Supplementary material for: A boosting method for maximizing the partial area under the ROC curve
Source: BMC Bioinformatics. 2010 Jun 10;11:314. doi: 10.1186/1471-2105-11-314 (PMC2898798; doi:10.1186/1471-2105-11-314)
Supplement: Additional file 1 — Details of the pAUCBoost algorithm. gives the details of the pAUCBoost algorithm. [file 1471-2105-11-314-S1.PDF]

Details of the pAUCBoost algorithm

In the step 1 of the pAUCBoost algorithm, we set the initial values of $F(\boldsymbol{x})$ and $\beta(f)$ for each weak classifier $f \in \mathcal{F}$. Then, for predetermined values of $\overline{\alpha}_1$ and $\overline{\alpha}_2$, we calculate the corresponding thresholds $\overline{c}_1$ and $\overline{c}_2$ for $F_{t-1} + \beta_{t-1}(f)$ in the step 2 (a). The first and second derivative of the objective function, which are used in the Newton-Raphson iteration, are given as

$$\begin{aligned}
&D_1(\beta_{t-1}(f)) \\
=&\frac{\partial}{\partial \beta_{t-1}(f)} \overline{\mathrm{pAUC}}_\lambda(F_{t-1} + \beta_{t-1}(f)f, \overline{\alpha}_1, \overline{\alpha}_2) \\
=&\frac{1}{n_0 n_1} \sum_{i \in I} \sum_{j \in J_{\mathrm{fan}}} \phi\Big(F_{t-1}(\boldsymbol{x}_{1j}) - F_{t-1}(\boldsymbol{x}_{0i}) + \beta_{t-1}(f)\{f(\boldsymbol{x}_{1j}) - f(\boldsymbol{x}_{0i})\}\Big)\Big(f(\boldsymbol{x}_{1j}) - f(\boldsymbol{x}_{0i})\Big) \\
&\quad + \frac{1}{n_0 n_1} \sum_{i \in I} \sum_{j \in J_{\mathrm{rec}}} \mathrm{I}\Big(F_{t-1}(\boldsymbol{x}_{1j}) - F_{t-1}(\boldsymbol{x}_{0i}) + \beta_{t-1}(f)\{f(\boldsymbol{x}_{1j}) - f(\boldsymbol{x}_{0i})\} = 0\Big)\Big(f(\boldsymbol{x}_{1j}) - f(\boldsymbol{x}_{0i})\Big) \\
&\quad - 2\lambda \int \big\{F''_{t-1,k}(x_k) + \beta_{t-1}(f) f''(\boldsymbol{x})\big\} f''_t(\boldsymbol{x}) dx_k,
\end{aligned}$$

and

$$\begin{aligned}
&D_2(\beta_{t-1}(f)) \\
=&\frac{\partial^2}{\partial \beta_{t-1}(f)^2} \overline{\mathrm{pAUC}}_\lambda(F_{t-1} + \beta_{t-1}(f)f, \overline{\alpha}_1, \overline{\alpha}_2) \\
=&-\frac{1}{n_0 n_1} \sum_{i \in I} \sum_{j \in J_{\mathrm{fan}}} \phi\Big(F_{t-1}(\boldsymbol{x}_{1j}) - F_{t-1}(\boldsymbol{x}_{0i}) + \beta_{t-1}(f)\{f(\boldsymbol{x}_{1j}) - f(\boldsymbol{x}_{0i})\}\Big) \\
&\quad \times \Big(F_{t-1}(\boldsymbol{x}_{1j}) - F_{t-1}(\boldsymbol{x}_{0i}) + \beta_{t-1}(f)\{f(\boldsymbol{x}_{1j}) - f(\boldsymbol{x}_{0i})\}\Big)\Big(f(\boldsymbol{x}_{1j}) - f(\boldsymbol{x}_{0i})\Big)^2 \\
&\quad - 2\lambda \int \big\{f''(\boldsymbol{x})\big\}^2 dx_k,
\end{aligned}$$

where $f(\boldsymbol{x})$ is one of $N_{k,l}(x_k)/Z_{k,l}$'s in $\mathcal{F}$, and the derivative of the Heaviside function H is replaced with the indicator function I. A basis function of natural cubic spline for x_k is explicitly given as

$$N_{k,l}(x_k) = \begin{cases} 1, \ l = 1, \\ x_k, \ l = 2, \\ d_{l-2}(x_k) - d_{m_k - 1}(x_k), \ \text{otherwise}, \end{cases}$$

where

$$d_l(x_k) = \frac{(x_k - \xi_{k,l-2})^3_+ - (x_k - \xi_{k,m_k})^3_+}{\xi_{k,m_k} - \xi_{k,l-2}},$$

where $\xi_{k,l}$ is one of m_k knots for x_k ($\xi_{k,1} < \xi_{k,2} < \ldots < \xi_{k,m_k}$), and z_+ denotes the positive part of z. The standardization factor $Z_{k,l}$ is given as

$$Z_{k,l} = \begin{cases} 1, \ l = 1, \\ \xi_{k,m_k} - \xi_{k,1}, \ l = 2, \\ N_{k,l}(\xi_{k,m_k}) - N_{k,l}(\xi_{k,l-2}), \ \text{otherwise}. \end{cases}$$

The knots are set to the observed values of x_k or the quantiles depending on the sample size and the number of the components of $\boldsymbol{x}$. We take a moderate number of quantiles for computational cost.

Then, we apply the Newton-Raphson method to get a set of coefficients at iteration number t:

$$\beta_t(f) = \beta_{t-1}(f) - \frac{D_1(\beta_{t-1}(f))}{D_2(\beta_{t-1}(f))}.$$

We observed that the value of $\beta_t(f)$ is unstable, especially when the cardinalities of I, J_{fan} and J_{rec} are very small. So, we restricted the maximum absolute value to be 1. Using the updated $\beta_t(f)$, the best weak classifier is chosen as

$$f_t = \underset{f}{\text{argmax}} \ \overline{\text{pAUC}}_\lambda(F_{t-1} + \beta_t(f)f, \overline{\alpha}_1, \overline{\alpha}_2). \tag{A.1}$$

The penalty term in Equation (A.1) for x_k is expressed as:

$$\begin{aligned} \int \Big\{F''_{t,k}(x_k)\Big\}^2 dx_k &= \int \Big\{ \sum_{l \in L_{k,t}} \beta_{k,l} N''_{k,l}(x_k)/Z_{k,l} \Big\}^2 dx_k \\ &= \sum_{l \in L_{k,t}} \sum_{l' \in L_{k,t}} \beta_{k,l}\beta_{k,l'} P_{k,l,l'}, \end{aligned} \tag{A.2}$$

where $L_{k,t}$ indicates a set of knots for x_k chosen until the iteration number t; $\beta_{k,l}$ is the coefficient for $N_{k,l}(x_k)/Z_{k,l}$. If $3 \le l \le l' \le m_k$, the term $P_{k,l,l'}$ is calculated as

$$\begin{aligned} &P_{k,l,l'} \\ &= \int N''_{k,l}(x_k) N''_{k,l'}(x_k)/(Z_{k,l}Z_{k,l'})dx_k \\ &= \int_{\xi_{k,l'-2}}^{\xi_{k,m_k-1}} N''_{k,l}(x_k)N''_{k,l'}(x_k)dx_k/(Z_{k,l}Z_{k,l'})dx_k + \int_{\xi_{k,m_k-1}}^{\xi_{k,m_k}} N''_{k,l}(x_k)N''_{k,l'}(x_k)dx_k/(Z_{k,l}Z_{k,l'})dx_k \\ &= \frac{6(\xi_{k,m_k-1} - \xi_{k,l'-2})\big\{(2\xi_{k,m_k-1} + \xi_{k,l'-2} - 3\xi_{k,l-2})(\xi_{k,m_k-1} - \xi_{k,l'-2}) + 2(\xi_{k,m_k} - \xi_{k,m_k-1})(\xi_{k,m_k-1} - \xi_{k,l-2})\big\}}{(\xi_{k,m_k} - \xi_{k,l-2})(\xi_{k,m_k} - \xi_{k,l'-2})Z_{k,l}Z_{k,l'}} \\ &= \frac{6\big\{2(\xi_{k,m_k} - \xi_{k,m_k-1})(\xi_{k,m_k-1} - \xi_{k,l-2}) + (2\xi_{k,m_k-1} + \xi_{k,l'-2} - 3\xi_{k,l-2})(\xi_{k,m_k-1} - \xi_{k,l'-2})\big\}}{(\xi_{k,m_k} - \xi_{k,l-2})(\xi_{k,m_k} - \xi_{k,l'-2})(2\xi_{k,m_k} - \xi_{k,m_k-1} - \xi_{k,l-2})(\xi_{k,m_k-1} - \xi_{k,l-2})(2\xi_{k,m_k} - \xi_{k,m_k-1} - \xi_{k,l'-2})}, \end{aligned}$$

otherwise ($l \le 2$ or $l' \le 2$) $P_{k,l,l'} = 0$.

Note that $P_{k,l,l'}$ depends on the scale of x_k. It means the penalty term also depends on the scale. Hence, by adjusting the scale, we make the maximum value of $P_{k,l,l'}$ in accordance for each k so that each x_k is penalized almost equally:

$$\max_{1 \le l, \ l' \le m_k} P_{k,l,l'} = P_{k,m_k,m_k} = 1.$$

On the other hand, in the case where x_k is discrete or categorical, we use a decision stump as a weak classifier:

$$S_{k,l}(x_k) = \mathrm{H}(x_k - \xi_{k,l}).$$

The corresponding penalty term for x_k is given as:

$$\sum_{x_k \in \Xi_k} \left\{ F_{t,k}^{(2)}(x_k) \right\}^2,$$

where $F_{t,k}^{(2)}(x_k)$ denotes the second-order difference of $F_{t,k}(x_k)$: $F_{t,k}^{(2)}(x_k) = F_{t,k}(x_k^{(-1)}) - 2F_{t,k}(x_k) + F_{t,k}(x_k^{(+1)})$ for $x_k^{(-1)} < x_k^{(+1)}$, and Ξ_k is a set of $\xi_{k,l}$ $(l = 1, 2, \ldots, m_k)$. In a similar manner to Equation (A.2), it is rewritten as:

$$\begin{aligned} \sum_{x_k \in \Xi_k} \left\{ F_{t,k}^{(2)}(x_k) \right\}^2 &= \sum_{x_k \in \Xi_k} \left\{ \sum_{l \in L_{k,t}} \beta_{k,l} S_{k,l}^{(2)}(x_k) \right\}^2 \\ &= \sum_{l \in L_{k,t}} \sum_{l' \in L_{k,t}} \beta_{k,l} \beta_{k,l'} Q_{k,l,l'}, \end{aligned}$$

where $\beta_{k,l}$ is the coefficient for $S_{k,l}(x_k)$, and

$$Q_{k,l,l'} = \sum_{x_k \in \Xi_k} S_{k,l}^{(2)}(x_k) S_{k,l'}^{(2)}(x_k) = \begin{cases} 2, \ l = l', \\ -1, \ |l - l'| = 1, \\ 0, \ |l - l'| \geq 2, \end{cases}$$

where $S_{k,l}^{(2)}(x_k)$ denotes the second-order difference of $S_{k,l}(x_k)$. The values of $Q_{k,l,l'}$ are necessary when we use decision stumps together with natural cubic splines as weak classifiers. See [12] for further details about the penalty for the decision stumps.
