# Supplementary material for: A boosting method for maximizing the partial area under the ROC curve
Source: BMC Bioinformatics. 2010 Jun 10;11:314. doi: 10.1186/1471-2105-11-314 (PMC2898798; doi:10.1186/1471-2105-11-314)
Supplement: Additional file 2 — Proof of Theorem 1 and Corollary 1. contains the details of the proof of Theorem 1 and Corollary 1. [file 1471-2105-11-314-S2.PDF]

Proof of Theorem 1 and Corollary 1

Proof of Theorem 1

At first, we fix the value of α_1 and α_2 using thresholds $c_{1,F}$ and $c_{2,F}$ as

$$\int \mathrm{H}(F(\boldsymbol{x}_0) - c_{1,F})g_0(\boldsymbol{x}_0)d\boldsymbol{x}_0 = \alpha_1, \ \int \mathrm{H}(F(\boldsymbol{x}_0) - c_{2,F})g_0(\boldsymbol{x}_0)d\boldsymbol{x}_0 = \alpha_2,$$

where $\alpha_1 < \alpha_2$ $(c_{2,F} < c_{1,F})$. For simplicity, we write

$$\begin{aligned}
\mathrm{H}_{F,i}(\boldsymbol{x}) &= \mathrm{H}(F(\boldsymbol{x}) - c_{i,F}), \\
\mathrm{H}_{i,F}(\boldsymbol{x}) &= \mathrm{H}(c_{i,F} - F(\boldsymbol{x})), \ i = 1, 2.
\end{aligned}$$

Then, the pAUC with FPR being between α_1 and α_2 has an integral formula:

$$\begin{aligned}
& \mathrm{pAUC}(F, \alpha_1, \alpha_2) \\
= & \int\int \mathrm{H}_{F,2}(\boldsymbol{x}_0)\mathrm{H}_{1,F}(\boldsymbol{x}_0)\mathrm{H}(F(\boldsymbol{x}_1) - F(\boldsymbol{x}_0))g_0(\boldsymbol{x}_0)g_1(\boldsymbol{x}_1)d\boldsymbol{x}_0 d\boldsymbol{x}_1 \\
= & \int\int \mathrm{H}_{F,2}(\boldsymbol{x}_0)\mathrm{H}_{1,F}(\boldsymbol{x}_0)\mathrm{H}_{F,2}(\boldsymbol{x}_1)\mathrm{H}(F(\boldsymbol{x}_1) - F(\boldsymbol{x}_0))g_0(\boldsymbol{x}_0)g_1(\boldsymbol{x}_1)d\boldsymbol{x}_0 d\boldsymbol{x}_1 \\
= & \int\int \mathrm{H}_{F,2}(\boldsymbol{x}_0)\mathrm{H}_{1,F}(\boldsymbol{x}_0)\mathrm{H}_{F,2}(\boldsymbol{x}_1)\Big\{\mathrm{H}_{1,F}(\boldsymbol{x}_1) + \mathrm{H}_{F,1}(\boldsymbol{x}_1)\Big\}\mathrm{H}(F(\boldsymbol{x}_1) - F(\boldsymbol{x}_0))g_0(\boldsymbol{x}_0)g_1(\boldsymbol{x}_1)d\boldsymbol{x}_0 d\boldsymbol{x}_1 \\
= & \int\int \mathrm{H}_{F,2}(\boldsymbol{x}_0)\mathrm{H}_{1,F}(\boldsymbol{x}_0)\mathrm{H}_{F,2}(\boldsymbol{x}_1)\mathrm{H}_{1,F}(\boldsymbol{x}_1)\mathrm{H}(F(\boldsymbol{x}_1) - F(\boldsymbol{x}_0))g_0(\boldsymbol{x}_0)g_1(\boldsymbol{x}_1)d\boldsymbol{x}_0 d\boldsymbol{x}_1 \\
& + \int\int \mathrm{H}_{F,2}(\boldsymbol{x}_0)\mathrm{H}_{1,F}(\boldsymbol{x}_0)\mathrm{H}_{F,2}(\boldsymbol{x}_1)\mathrm{H}_{F,1}(\boldsymbol{x}_1)\mathrm{H}(F(\boldsymbol{x}_1) - F(\boldsymbol{x}_0))g_0(\boldsymbol{x}_0)g_1(\boldsymbol{x}_1)d\boldsymbol{x}_0 d\boldsymbol{x}_1 \\
= & \int\int \mathrm{H}_{F,2}(\boldsymbol{x}_0)\mathrm{H}_{1,F}(\boldsymbol{x}_0)\mathrm{H}_{F,2}(\boldsymbol{x}_1)\mathrm{H}_{1,F}(\boldsymbol{x}_1)\mathrm{H}(F(\boldsymbol{x}_1) - F(\boldsymbol{x}_0))g_0(\boldsymbol{x}_0)g_1(\boldsymbol{x}_1)d\boldsymbol{x}_0 d\boldsymbol{x}_1 \\
& + \int \mathrm{H}_{F,2}(\boldsymbol{x}_0)\mathrm{H}_{1,F}(\boldsymbol{x}_0)g_0(\boldsymbol{x}_0)d\boldsymbol{x}_0 \int \mathrm{H}_{F,1}(\boldsymbol{x}_1)g_1(\boldsymbol{x}_1)d\boldsymbol{x}_1.
\end{aligned}$$

Similarly, the approximate pAUC is given as

$$\begin{aligned}
& \mathrm{pAUC}_\sigma(F, \alpha_1, \alpha_2) \\
= & \int\int \mathrm{H}_{F,2}(\boldsymbol{x}_0)\mathrm{H}_{1,F}(\boldsymbol{x}_0)\mathrm{H}_{F,2}(\boldsymbol{x}_1)\mathrm{H}_{1,F}(\boldsymbol{x}_1)\mathrm{H}_\sigma(F(\boldsymbol{x}_1) - F(\boldsymbol{x}_0))g_0(\boldsymbol{x}_0)g_1(\boldsymbol{x}_1)d\boldsymbol{x}_0 d\boldsymbol{x}_1 \\
& + \int \mathrm{H}_{F,2}(\boldsymbol{x}_0)\mathrm{H}_{1,F}(\boldsymbol{x}_0)g_0(\boldsymbol{x}_0)d\boldsymbol{x}_0 \int \mathrm{H}_{F,1}(\boldsymbol{x}_1)g_1(\boldsymbol{x}_1)d\boldsymbol{x}_1.
\end{aligned}$$

Note that the domain of integration can be restricted to $\mathcal{X} = \{\boldsymbol{x} | g_0(\boldsymbol{x}) \neq 0, g_1(\boldsymbol{x}) \neq 0\}$ without loss of generality. Hence, we will prove Theorem 1 based on this restricted domain; though, we omit the notation for simplicity.

Proof. For simplicity, we define some notations:

$$\begin{aligned}\zeta(\boldsymbol{x}) &= m\big(\Lambda(\boldsymbol{x})\big),\\ F_{\gamma\zeta}(\boldsymbol{x}) &= F(\boldsymbol{x}) + \gamma\zeta(\boldsymbol{x})\\ c'_{i,F_{\gamma\zeta}} &= \frac{\partial c_{i,F_{\gamma\zeta}}}{\partial\gamma},\ i=1,2.\end{aligned}$$

Then, the first derivative of $\Psi(\gamma)$ with respect to γ is given as

$$\begin{aligned}&\frac{\partial}{\partial\gamma}\Psi(\gamma)\\ =\ & \int\int \mathrm{H}'(F_{\gamma\zeta}(\boldsymbol{x}_0) - c_{2,F_{\gamma\zeta}})(\zeta(\boldsymbol{x}_0) - c'_{2,F_{\gamma\zeta}})\mathrm{H}_{1,F_{\gamma\zeta}}(\boldsymbol{x}_0)\mathrm{H}_{F_{\gamma\zeta},2}(\boldsymbol{x}_1)\\ &\quad\times \mathrm{H}_{1,F_{\gamma\zeta}}(\boldsymbol{x}_1)\mathrm{H}_\sigma(F_{\gamma\zeta}(\boldsymbol{x}_1) - F_{\gamma\zeta}(\boldsymbol{x}_0))g_0(\boldsymbol{x}_0)g_1(\boldsymbol{x}_1)d\boldsymbol{x}_0 d\boldsymbol{x}_1\\ &+\int\int \mathrm{H}_{F_{\gamma\zeta},2}(\boldsymbol{x}_0)\mathrm{H}'(c_{1,F_{\gamma\zeta}} - F_{\gamma\zeta}(\boldsymbol{x}_0))(c'_{1,F_{\gamma\zeta}} - \zeta(\boldsymbol{x}_0))\mathrm{H}_{F_{\gamma\zeta},2}(\boldsymbol{x}_1)\\ &\quad\times \mathrm{H}_{1,F_{\gamma\zeta}}(\boldsymbol{x}_1)\mathrm{H}_\sigma(F_{\gamma\zeta}(\boldsymbol{x}_1) - F_{\gamma\zeta}(\boldsymbol{x}_0))g_0(\boldsymbol{x}_0)g_1(\boldsymbol{x}_1)d\boldsymbol{x}_0 d\boldsymbol{x}_1\\ &+\int\int \mathrm{H}_{F_{\gamma\zeta},2}(\boldsymbol{x}_0)\mathrm{H}_{1,F_{\gamma\zeta}}(\boldsymbol{x}_0)\mathrm{H}'(F_{\gamma\zeta}(\boldsymbol{x}_1) - c_{2,F_{\gamma\zeta}})(\zeta(\boldsymbol{x}_1) - c'_{2,F_{\gamma\zeta}})\\ &\quad\times \mathrm{H}_{1,F_{\gamma\zeta}}(\boldsymbol{x}_1)\mathrm{H}_\sigma(F_{\gamma\zeta}(\boldsymbol{x}_1) - F_{\gamma\zeta}(\boldsymbol{x}_0))g_0(\boldsymbol{x}_0)g_1(\boldsymbol{x}_1)d\boldsymbol{x}_0 d\boldsymbol{x}_1\\ &+\int\int \mathrm{H}_{F_{\gamma\zeta},2}(\boldsymbol{x}_0)\mathrm{H}_{1,F_{\gamma\zeta}}(\boldsymbol{x}_0)\mathrm{H}_{F_{\gamma\zeta},2}(\boldsymbol{x}_1)\\ &\quad\times \mathrm{H}'(c_{1,F_{\gamma\zeta}} - F_{\gamma\zeta}(\boldsymbol{x}_1))(c'_{1,F_{\gamma\zeta}} - \zeta(\boldsymbol{x}_1))\mathrm{H}_\sigma(F_{\gamma\zeta}(\boldsymbol{x}_1) - F_{\gamma\zeta}(\boldsymbol{x}_0))g_0(\boldsymbol{x}_0)g_1(\boldsymbol{x}_1)d\boldsymbol{x}_0 d\boldsymbol{x}_1\\ &+\int\int \mathrm{H}_{F_{\gamma\zeta},2}(\boldsymbol{x}_0)\mathrm{H}_{1,F_{\gamma\zeta}}(\boldsymbol{x}_0)\mathrm{H}_{F_{\gamma\zeta},2}(\boldsymbol{x}_1)\\ &\quad\times \mathrm{H}_{1,F_{\gamma\zeta}}(\boldsymbol{x}_1)\mathrm{H}'_\sigma(F_{\gamma\zeta}(\boldsymbol{x}_1) - F_{\gamma\zeta}(\boldsymbol{x}_0))(\zeta(\boldsymbol{x}_1) - \zeta(\boldsymbol{x}_0))g_0(\boldsymbol{x}_0)g_1(\boldsymbol{x}_1)d\boldsymbol{x}_0 d\boldsymbol{x}_1\\ &+\int\Big\{\mathrm{H}'(F_{\gamma\zeta}(\boldsymbol{x}_0) - c_{2,F_{\gamma\zeta}})(\zeta(\boldsymbol{x}_0) - c'_{2,F_{\gamma\zeta}}) - \mathrm{H}'_{F_{\gamma\zeta},1}(\boldsymbol{x}_0)(\zeta(\boldsymbol{x}_0) - c'_{1,F_{\gamma\zeta}})\Big\}g_0(\boldsymbol{x}_0)d\boldsymbol{x}_0\mathrm{TPR}(F_{\gamma\zeta}, c_{1,F_{\gamma\zeta}})\\ &+\int \mathrm{H}_{F_{\gamma\zeta},2}(\boldsymbol{x}_0)\mathrm{H}_{1,F_{\gamma\zeta}}(\boldsymbol{x}_0)g_0(\boldsymbol{x}_0)d\boldsymbol{x}_0\mathrm{TPR}'(F_{\gamma\zeta}, c_{1,F_{\gamma\zeta}}),\end{aligned}$$

where

$$\mathrm{TPR}(F_{\gamma\zeta}, c_{1,F_{\gamma\zeta}}) = \int \mathrm{H}_{F_{\gamma\zeta},1}(\boldsymbol{x}_1)g_1(\boldsymbol{x}_1)d\boldsymbol{x}_1.$$

And the first derivative is rewritten such as

$$
\begin{aligned}
&\frac{\partial}{\partial\gamma}\Psi(\gamma)\\
=&\int \mathrm{H}'(F_{\gamma\zeta}(\boldsymbol{x}_0)-c_{2,F_{\gamma\zeta}})(\zeta(\boldsymbol{x}_0)-c'_{2,F_{\gamma\zeta}})g_0(\boldsymbol{x}_0)d\boldsymbol{x}_0\int \mathrm{H}_{F_{\gamma\zeta},2}(\boldsymbol{x}_1)\mathrm{H}_{1,F_{\gamma\zeta}}(\boldsymbol{x}_1)\mathrm{H}_\sigma(F_{\gamma\zeta}(\boldsymbol{x}_1)-c_{2,F_{\gamma\zeta}})g_1(\boldsymbol{x}_1)d\boldsymbol{x}_1\\
&+\int \mathrm{H}'(c_{1,F_{\gamma\zeta}}-F_{\gamma\zeta}(\boldsymbol{x}_0))(c'_{1,F_{\gamma\zeta}}-\zeta(\boldsymbol{x}_0))g_0(\boldsymbol{x}_0)d\boldsymbol{x}_0\int \mathrm{H}_{F_{\gamma\zeta},2}(\boldsymbol{x}_1)\mathrm{H}_{1,F_{\gamma\zeta}}(\boldsymbol{x}_1)\mathrm{H}_\sigma(F_{\gamma\zeta}(\boldsymbol{x}_1)-c_{1,F_{\gamma\zeta}})g_1(\boldsymbol{x}_1)d\boldsymbol{x}_1\\
&+\int \mathrm{H}'(F_{\gamma\zeta}(\boldsymbol{x}_1)-c_{2,F_{\gamma\zeta}})(\zeta(\boldsymbol{x}_1)-c'_{2,F_{\gamma\zeta}})g_1(\boldsymbol{x}_1)d\boldsymbol{x}_1\int \mathrm{H}_{F_{\gamma\zeta},2}(\boldsymbol{x}_0)\mathrm{H}_{1,F_{\gamma\zeta}}(\boldsymbol{x}_0)\mathrm{H}_\sigma(c_{2,F_{\gamma\zeta}}-F_{\gamma\zeta}(\boldsymbol{x}_0))g_0(\boldsymbol{x}_0)d\boldsymbol{x}_0\\
&+\int \mathrm{H}'(c_{1,F_{\gamma\zeta}}-F_{\gamma\zeta}(\boldsymbol{x}_1))(c'_{1,F_{\gamma\zeta}}-\zeta(\boldsymbol{x}_1))g_1(\boldsymbol{x}_1)d\boldsymbol{x}_1\int \mathrm{H}_{F_{\gamma\zeta},2}(\boldsymbol{x}_0)\mathrm{H}_{1,F_{\gamma\zeta}}(\boldsymbol{x}_0)\mathrm{H}_\sigma(c_{1,F_{\gamma\zeta}}-F_{\gamma\zeta}(\boldsymbol{x}_0))g_0(\boldsymbol{x}_0)d\boldsymbol{x}_0\\
&+\int\int \mathrm{H}_{F_{\gamma\zeta},2}(\boldsymbol{x}_0)\mathrm{H}_{1,F_{\gamma\zeta}}(\boldsymbol{x}_0)\mathrm{H}_{F_{\gamma\zeta},2}(\boldsymbol{x}_1)\mathrm{H}_{1,F_{\gamma\zeta}}(\boldsymbol{x}_1)\mathrm{H}'_\sigma(F_{\gamma\zeta}(\boldsymbol{x}_1)-F_{\gamma\zeta}(\boldsymbol{x}_0))(\zeta(\boldsymbol{x}_1)-\zeta(\boldsymbol{x}_0))g_0(\boldsymbol{x}_0)g_1(\boldsymbol{x}_1)d\boldsymbol{x}_0d\boldsymbol{x}_1\\
&+\int\Big\{\mathrm{H}'(F_{\gamma\zeta}(\boldsymbol{x}_0)-c_{2,F_{\gamma\zeta}})(\zeta(\boldsymbol{x}_0)-c'_{2,F_{\gamma\zeta}})-\mathrm{H}'(F_{\gamma\zeta}(\boldsymbol{x}_0)-c_{1,F_{\gamma\zeta}})(\zeta(\boldsymbol{x}_0)-c'_{1,F_{\gamma\zeta}})\Big\}g_0(\boldsymbol{x}_0)d\boldsymbol{x}_0\mathrm{TPR}(F_{\gamma\zeta},c_{1,F_{\gamma\zeta}})\\
&+\int \mathrm{H}_{F_{\gamma\zeta},2}(\boldsymbol{x}_0)\mathrm{H}_{1,F_{\gamma\zeta}}(\boldsymbol{x}_0)g_0(\boldsymbol{x}_0)d\boldsymbol{x}_0\mathrm{TPR}'(F_{\gamma\zeta},c_{1,F_{\gamma\zeta}}),\\
=&\mathrm{FPR}'(F_{\gamma\zeta},c_{2,F_{\gamma\zeta}})\int \mathrm{H}_{F_{\gamma\zeta},2}(\boldsymbol{x}_1)\mathrm{H}_{1,F_{\gamma\zeta}}(\boldsymbol{x}_1)\mathrm{H}_\sigma(F_{\gamma\zeta}(\boldsymbol{x}_1)-c_{2,F_{\gamma\zeta}})g_1(\boldsymbol{x}_1)d\boldsymbol{x}_1\\
&-\mathrm{FPR}'(F_{\gamma\zeta},c_{1,F_{\gamma\zeta}})\int \mathrm{H}_{F_{\gamma\zeta},2}(\boldsymbol{x}_1)\mathrm{H}_{1,F_{\gamma\zeta}}(\boldsymbol{x}_1)\mathrm{H}_\sigma(F_{\gamma\zeta}(\boldsymbol{x}_1)-c_{1,F_{\gamma\zeta}})g_1(\boldsymbol{x}_1)d\boldsymbol{x}_1\\
&+\mathrm{TPR}'(F_{\gamma\zeta},c_{2,F_{\gamma\zeta}})\int \mathrm{H}_{F_{\gamma\zeta},2}(\boldsymbol{x}_0)\mathrm{H}_{1,F_{\gamma\zeta}}(\boldsymbol{x}_0)\mathrm{H}_\sigma(c_{2,F_{\gamma\zeta}}-F_{\gamma\zeta}(\boldsymbol{x}_0))g_0(\boldsymbol{x}_0)d\boldsymbol{x}_0\\
&-\mathrm{TPR}'(F_{\gamma\zeta},c_{1,F_{\gamma\zeta}})\int \mathrm{H}_{F_{\gamma\zeta},2}(\boldsymbol{x}_0)\mathrm{H}_{1,F_{\gamma\zeta}}(\boldsymbol{x}_0)\mathrm{H}_\sigma(c_{1,F_{\gamma\zeta}}-F_{\gamma\zeta}(\boldsymbol{x}_0))g_0(\boldsymbol{x}_0)d\boldsymbol{x}_0\\
&+\int\int \mathrm{H}_{F_{\gamma\zeta},2}(\boldsymbol{x}_0)\mathrm{H}_{1,F_{\gamma\zeta}}(\boldsymbol{x}_0)\mathrm{H}_{F_{\gamma\zeta},2}(\boldsymbol{x}_1)\mathrm{H}_{1,F_{\gamma\zeta}}(\boldsymbol{x}_1)\mathrm{H}'_\sigma(F_{\gamma\zeta}(\boldsymbol{x}_1)-F_{\gamma\zeta}(\boldsymbol{x}_0))(\zeta(\boldsymbol{x}_1)-\zeta(\boldsymbol{x}_0))g_0(\boldsymbol{x}_0)g_1(\boldsymbol{x}_1)d\boldsymbol{x}_0d\boldsymbol{x}_1\\
&+\Big\{\mathrm{FPR}'(F_{\gamma\zeta},c_{2,F_{\gamma\zeta}})-\mathrm{FPR}'(F_{\gamma\zeta},c_{1,F_{\gamma\zeta}})\Big\}\mathrm{TPR}(F_{\gamma\zeta},c_{1,F_{\gamma\zeta}})\\
&+\mathrm{TPR}'(F_{\gamma\zeta},c_{1,F_{\gamma\zeta}})\int \mathrm{H}_{F_{\gamma\zeta},2}(\boldsymbol{x}_0)\mathrm{H}_{1,F_{\gamma\zeta}}(\boldsymbol{x}_0)g_0(\boldsymbol{x}_0)d\boldsymbol{x}_0.
\end{aligned}
$$

Since $\mathrm{FPR}(F_{\gamma\zeta},c_{1,F_{\gamma\zeta}})$ and $\mathrm{FPR}(F_{\gamma\zeta},c_{2,F_{\gamma\zeta}})$ are fixed, we have

$$
\begin{aligned}
&\frac{\partial}{\partial\gamma}\Psi(\gamma)\\
=\;&\mathrm{TPR}'(F_{\gamma\zeta},c_{2,F_{\gamma\zeta}})\int \mathrm{H}_{F_{\gamma\zeta},2}(\boldsymbol{x}_0)\mathrm{H}_{1,F_{\gamma\zeta}}(\boldsymbol{x}_0)\mathrm{H}_\sigma(c_{2,F_{\gamma\zeta}}-F_{\gamma\zeta}(\boldsymbol{x}_0))g_0(\boldsymbol{x}_0)d\boldsymbol{x}_0\\
&+\mathrm{TPR}'(F_{\gamma\zeta},c_{1,F_{\gamma\zeta}})\int \mathrm{H}_{F_{\gamma\zeta},2}(\boldsymbol{x}_0)\mathrm{H}_{1,F_{\gamma\zeta}}(\boldsymbol{x}_0)\Big\{1-\mathrm{H}_\sigma(c_{1,F_{\gamma\zeta}}-F_{\gamma\zeta}(\boldsymbol{x}_0))\Big\}g_0(\boldsymbol{x}_0)d\boldsymbol{x}_0\\
&+\int\int \mathrm{H}_{F_{\gamma\zeta},2}(\boldsymbol{x}_0)\mathrm{H}_{1,F_{\gamma\zeta}}(\boldsymbol{x}_0)\mathrm{H}_{F_{\gamma\zeta},2}(\boldsymbol{x}_1)\\
&\quad\times\mathrm{H}_{1,F_{\gamma\zeta}}(\boldsymbol{x}_1)\mathrm{H}'_\sigma(F_{\gamma\zeta}(\boldsymbol{x}_1)-F_{\gamma\zeta}(\boldsymbol{x}_0))(\zeta(\boldsymbol{x}_1)-\zeta(\boldsymbol{x}_0))g_0(\boldsymbol{x}_0)g_1(\boldsymbol{x}_1)d\boldsymbol{x}_0d\boldsymbol{x}_1. \qquad \text{(B.1)}
\end{aligned}
$$

Next, we investigate the behavior of $\mathrm{TPR}'(F_{\gamma\zeta},c_{2,F_{\gamma\zeta}})$. The value of $\mathrm{FPR}(F_{\gamma\zeta},c_{2,F_{\gamma\zeta}})$ is fixed, so we have

$$
\mathrm{FPR}'(F_{\gamma\zeta},c_{2,F_{\gamma\zeta}})=\int \mathrm{H}'(F_{\gamma\zeta}(\boldsymbol{x}_0)-c_{2,F_{\gamma\zeta}})(\zeta(\boldsymbol{x}_0)-c'_{2,F_{\gamma\zeta}})g_0(\boldsymbol{x}_0)d\boldsymbol{x}_0=0.
$$

Then we have

$$c'_{2,F_{\gamma\zeta}} = \frac{\int \mathrm{H}'(F_{\gamma\zeta}(\boldsymbol{x}_0) - c_{2,F_{\gamma\zeta}})\zeta(\boldsymbol{x}_0)g_0(\boldsymbol{x}_0)d\boldsymbol{x}_0}{\int \mathrm{H}'(F_{\gamma\zeta}(\boldsymbol{x}_0) - c_{2,F_{\gamma\zeta}})g_0(\boldsymbol{x}_0)d\boldsymbol{x}_0},$$

where the denominator is not zero because the domain of integration is $\mathcal{X} = \{\boldsymbol{x}|g_0(\boldsymbol{x}) \neq 0, g_1(\boldsymbol{x}) \neq 0\}$. By substituting it into $\mathrm{TPR}'(F_{\gamma\zeta}, c_{2,F_{\gamma\zeta}})$, we have

$$\begin{aligned} & \mathrm{TPR}'(F_{\gamma\zeta}, c_{2,F_{\gamma\zeta}}) \\ = \;& \frac{\int\int K(\boldsymbol{x}_0,\boldsymbol{x}_1)\zeta(\boldsymbol{x}_1)g_0(\boldsymbol{x}_0)g_1(\boldsymbol{x}_1)d\boldsymbol{x}_0 d\boldsymbol{x}_1 - \int\int K(\boldsymbol{x}_0,\boldsymbol{x}_1)\zeta(\boldsymbol{x}_0)g_0(\boldsymbol{x}_0)g_1(\boldsymbol{x}_1)d\boldsymbol{x}_0 d\boldsymbol{x}_1}{\int \mathrm{H}'(F_{\gamma\zeta}(\boldsymbol{x}_0) - c_{2,F_{\gamma\zeta}})g_0(\boldsymbol{x}_0)d\boldsymbol{x}_0}, \end{aligned} \tag{B.2}$$

where

$$K(\boldsymbol{x}_0,\boldsymbol{x}_1) = \mathrm{H}'(F_{\gamma\zeta}(\boldsymbol{x}_0) - c_{2,F_{\gamma\zeta}})\mathrm{H}'(F_{\gamma\zeta}(\boldsymbol{x}_1) - c_{2,F_{\gamma\zeta}})$$

Then, the numerator becomes

$$\begin{aligned} & \int\int K(\boldsymbol{x}_0,\boldsymbol{x}_1)\Big(\zeta(\boldsymbol{x}_1) - \zeta(\boldsymbol{x}_0)\Big)g_0(\boldsymbol{x}_0)g_1(\boldsymbol{x}_1)d\boldsymbol{x}_0 d\boldsymbol{x}_1 \\ = \;& \int\int K(\boldsymbol{x}_1,\boldsymbol{x}_0)\Big(\zeta(\boldsymbol{x}_0) - \zeta(\boldsymbol{x}_1)\Big)g_0(\boldsymbol{x}_1)g_1(\boldsymbol{x}_0)d\boldsymbol{x}_1 d\boldsymbol{x}_0 \\ = \;& \frac{1}{2}\int\int K(\boldsymbol{x}_0,\boldsymbol{x}_1)\Big(\zeta(\boldsymbol{x}_1) - \zeta(\boldsymbol{x}_0)\Big)\Big(g_0(\boldsymbol{x}_0)g_1(\boldsymbol{x}_1) - g_0(\boldsymbol{x}_1)g_1(\boldsymbol{x}_0)\Big)d\boldsymbol{x}_0 d\boldsymbol{x}_1 \\ = \;& \frac{1}{2}\int\int K(\boldsymbol{x}_0,\boldsymbol{x}_1)\Big(\zeta(\boldsymbol{x}_1) - \zeta(\boldsymbol{x}_0)\Big)\Big(\Lambda(\boldsymbol{x}_1) - \Lambda(\boldsymbol{x}_0)\Big)g_0(\boldsymbol{x}_0)g_0(\boldsymbol{x}_1)d\boldsymbol{x}_0 d\boldsymbol{x}_1 \\ > \;& 0. \end{aligned} \tag{B.3}$$

Hence, we have

$$\mathrm{TPR}'(F_{\gamma\zeta}, c_{i,\gamma}) \geq 0,\; i = 1, 2,$$

because we can replace $c_{2,F_{\gamma\zeta}}$ with $c_{1,F_{\gamma\zeta}}$, and have the same result.

By looking at the third term in Equation (B.1), we find

$$\mathrm{H}_{F_{\gamma\zeta},2}(\boldsymbol{x}_0)\mathrm{H}_{1,F_{\gamma\zeta}}(\boldsymbol{x}_0)\mathrm{H}_{F_{\gamma\zeta},2}(\boldsymbol{x}_1)\mathrm{H}_{1,F_{\gamma\zeta}}(\boldsymbol{x}_1)\mathrm{H}'_\sigma(F_{\gamma\zeta}(\boldsymbol{x}_1) - F_{\gamma\zeta}(\boldsymbol{x}_0))$$

is invariant to the exchange of $\boldsymbol{x}_0$ for $\boldsymbol{x}_1$ like $K(\boldsymbol{x}_0, \boldsymbol{x}_1)$. Hence by the same argument above, we have

$$\begin{aligned}
& \int\int \mathrm{H}_{F_{\gamma\zeta},2}(\boldsymbol{x}_0)\mathrm{H}_{1,F_{\gamma\zeta}}(\boldsymbol{x}_0)\mathrm{H}_{F_{\gamma\zeta},2}(\boldsymbol{x}_1) \\
& \quad \times \mathrm{H}_{1,F_{\gamma\zeta}}(\boldsymbol{x}_1)\mathrm{H}'_\sigma(F_{\gamma\zeta}(\boldsymbol{x}_1) - F_{\gamma\zeta}(\boldsymbol{x}_0))\Big(\zeta(\boldsymbol{x}_1) - \zeta(\boldsymbol{x}_0)\Big)g_0(\boldsymbol{x}_0)g_1(\boldsymbol{x}_1)d\boldsymbol{x}_0 d\boldsymbol{x}_1 \\
= & \frac{1}{2}\int\int \mathrm{H}_{F_{\gamma\zeta},2}(\boldsymbol{x}_0)\mathrm{H}_{1,F_{\gamma\zeta}}(\boldsymbol{x}_0)\mathrm{H}_{F_{\gamma\zeta},2}(\boldsymbol{x}_1)\mathrm{H}_{1,F_{\gamma\zeta}}(\boldsymbol{x}_1)\mathrm{H}'_\sigma(F_{\gamma\zeta}(\boldsymbol{x}_1) - F_{\gamma\zeta}(\boldsymbol{x}_0))\Big(\zeta(\boldsymbol{x}_1) - \zeta(\boldsymbol{x}_0)\Big) \\
& \quad \times \Big(\Lambda(\boldsymbol{x}_1) - \Lambda(\boldsymbol{x}_0)\Big)g_0(\boldsymbol{x}_0)g_0(\boldsymbol{x}_1)d\boldsymbol{x}_0 d\boldsymbol{x}_1 \\
> & \; 0.
\end{aligned}$$

As a result, we have

$$\frac{\partial}{\partial\gamma}\Psi(\gamma) > 0.$$

Finally, we have

$$\begin{aligned}
\mathrm{pAUC}_\sigma(F, \alpha_1, \alpha_2) & < \lim_{\gamma\to\infty}\Psi(\gamma) \\
& = \lim_{\gamma\to\infty}\mathrm{pAUC}_\sigma\left[\gamma\left\{\frac{F}{\gamma} + \zeta\right\}, \alpha_1, \alpha_2\right] \\
& = \lim_{\gamma\to\infty}\mathrm{pAUC}_{\frac{\sigma}{\gamma}}\left(\frac{F}{\gamma} + \zeta, \alpha_1, \alpha_2\right) \\
& = \mathrm{pAUC}(\zeta, \alpha_1, \alpha_2) \\
& = \mathrm{pAUC}(\Lambda, \alpha_1, \alpha_2).
\end{aligned}$$

Since the inequation above holds for any F, $\lim_{\gamma\to\infty}\Psi(\gamma)$ is an upper bound of $\mathrm{pAUC}_\sigma(F, \alpha_1, \alpha_2)$. On the other hand, from the definition of the supremum of $\mathrm{pAUC}_\sigma(F, \alpha_1, \alpha_2)$ we have

$$\Psi(\gamma) = \mathrm{pAUC}_\sigma\Big(F + \gamma\, m\big(\Lambda\big), \alpha_1, \alpha_2\Big) \leq \sup_F \mathrm{pAUC}_\sigma(F, \alpha_1, \alpha_2).$$

By taking the limit of $\Psi(\gamma)$ as γ approaches ∞, we have

$$\lim_{\gamma\to\infty}\Psi(\gamma) \leq \sup_F \mathrm{pAUC}_\sigma(F, \alpha_1, \alpha_2).$$

As a result, we have

$$\lim_{\gamma\to\infty}\Psi(\gamma) = \sup_F \mathrm{pAUC}_\sigma(F, \alpha_1, \alpha_2),$$

which proves Theorem 1.

Proof of Corollary 1

At first we fix the value of FPR as $\mathrm{FPR}(F_{\gamma\eta}, c_{F_{\gamma\eta}}) = \alpha$. Then, the first derivative of $\mathrm{TPR}(F_{\gamma\eta}, c_{F_{\gamma\eta}})$ regarding to γ is given from (B.2) and (B.3) as

$$\begin{aligned} & \mathrm{TPR}'(F_{\gamma\eta}, c_{F_{\gamma\eta}}) \\ = \ & \frac{1}{2}\int\int K^*(\boldsymbol{x}_0, \boldsymbol{x}_1)\Big(\eta(\boldsymbol{x}_1) - \eta(\boldsymbol{x}_0)\Big)\Big(\Lambda(\boldsymbol{x}_1) - \Lambda(\boldsymbol{x}_0)\Big) g_0(\boldsymbol{x}_0) g_0(\boldsymbol{x}_1) d\boldsymbol{x}_0 d\boldsymbol{x}_1 \\ & \qquad \Big/ \int \mathrm{H}'(F_{\gamma\eta}(\boldsymbol{x}_0) - c_{F_{\gamma\eta}}) g_0(\boldsymbol{x}_0) d\boldsymbol{x}_0, \end{aligned}$$

where

$$K^*(\boldsymbol{x}_0, \boldsymbol{x}_1) = \mathrm{H}'(F_{\gamma\eta}(\boldsymbol{x}_0) - c_{F_{\gamma\eta}})\mathrm{H}'(F_{\gamma\eta}(\boldsymbol{x}_1) - c_{F_{\gamma\eta}}),$$

and the denominator is not zero because the domain of integration is $\mathcal{X} = \{\boldsymbol{x} | g_0(\boldsymbol{x}) \neq 0, g_1(\boldsymbol{x}) \neq 0\}$. The domain of the integration in the numerator is determined by K^*, which is dependent on an arbitrary score function F. Hence for any F, $\mathrm{TPR}'(F_{\gamma\eta}, c_{F_{\gamma\eta}}) \geq 0$ only if $\eta = m(\Lambda)$, where m is a strictly increasing function. The sufficiency is confirmed easily.
