# Supplementary material for: A boosting method for maximizing the partial area under the ROC curve
Source: BMC Bioinformatics. 2010 Jun 10;11:314. doi: 10.1186/1471-2105-11-314 (PMC2898798; doi:10.1186/1471-2105-11-314)
Supplement: Additional file 3 — Supplementary results of breast cancer data analysis. describes the supplementary results of breast cancer data, where the range of FPR is more relaxed. [file 1471-2105-11-314-S3.PDF]

The supplementary results of breast cancer data analysis

The results of other boosting methods are shown in Figure 1A. As a whole, they have tendencies to have smaller values of the pAUC regarding test data. However, AUCBoost and AdaBoost showed comparable performance with that of pAUCBoost at the small number of iterations. So, we conducted 10-fold cross validations for the two methods, and obtained $T = 40$ and $T = 10$, respectively. Hence, the resultant values of the pAUC turned out to be less than 0.04 for both methods.

Regarding pAUCBoost, we conducted the same analyses for gene expression data using different values of $\overline{\alpha}_2 = 0.2,\ 0.3$ with $\overline{\alpha}_1 = 0$. The selected genes based on the probability of gene selection proposed by Pepe et al. [27] are shown in Table 1A. The score plots for $\overline{\alpha}_2 = 0.2$ and $\overline{\alpha}_2 = 0.3$ are described in Figure 2A and Figure 3A, respectively. The resultant pAUC for training data and test data are 0.193, 0.05 for $\overline{\alpha}_2 = 0.2$; 0.278, 0.138 for $\overline{\alpha}_2 = 0.3$, respectively. The corresponding values by the method of van't Veer et al. [31] are 0.10, 0.017; 0.19, 0.16, respectively.

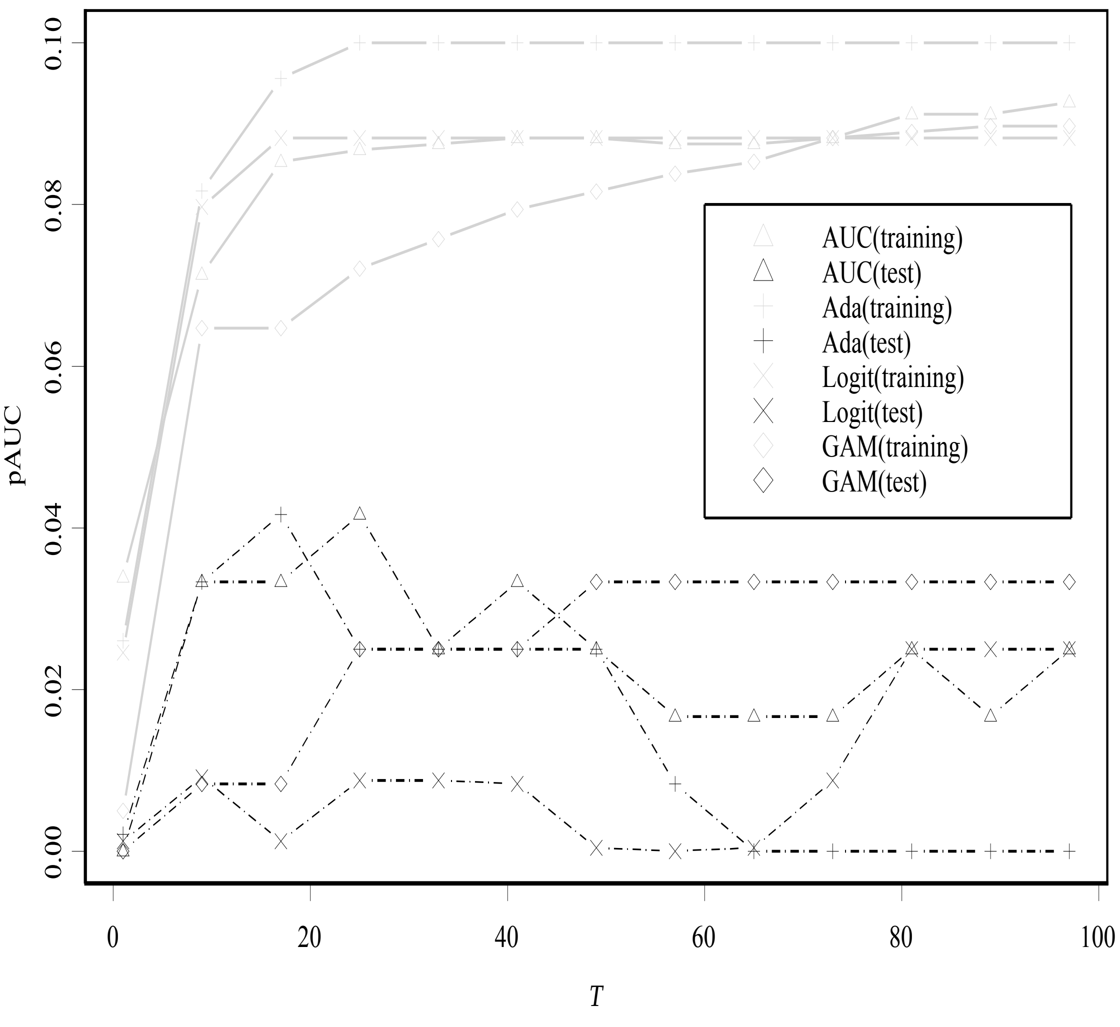

Figure 1A. Results of other boosting methods than pAUCBoost. The solid lines indicate the values of the pAUC based on test data; the gray lines based on training data.

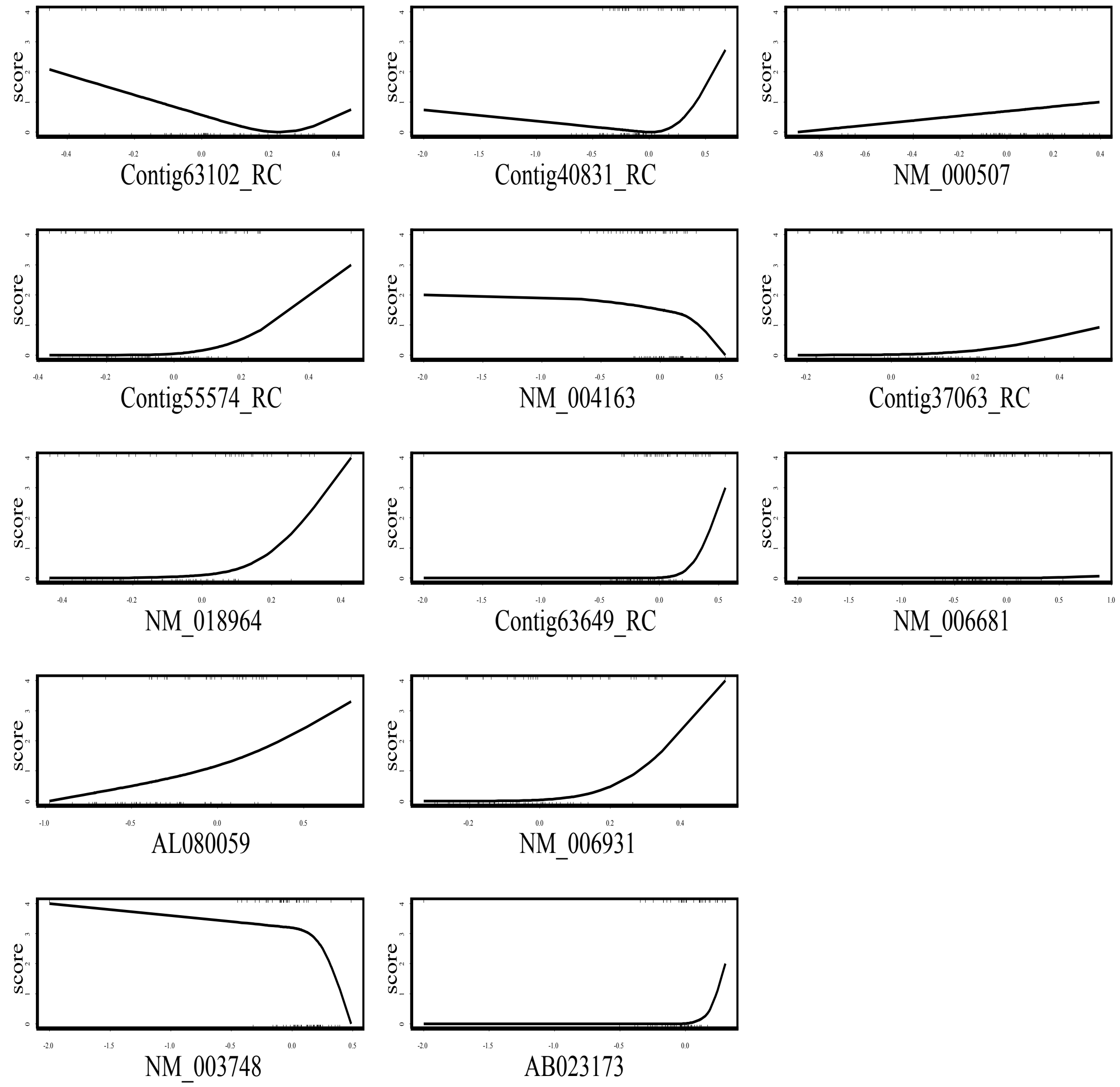

Figure 2A. Score plots of the 13 genes generated by pAUCBoost with $\overline{\alpha}_2 = 0.2$. The rug plot at the bottoms of each score plot shows observations of patients with good prognosis; the rug plot for patients with distant metastases is described at the top of each score plot.

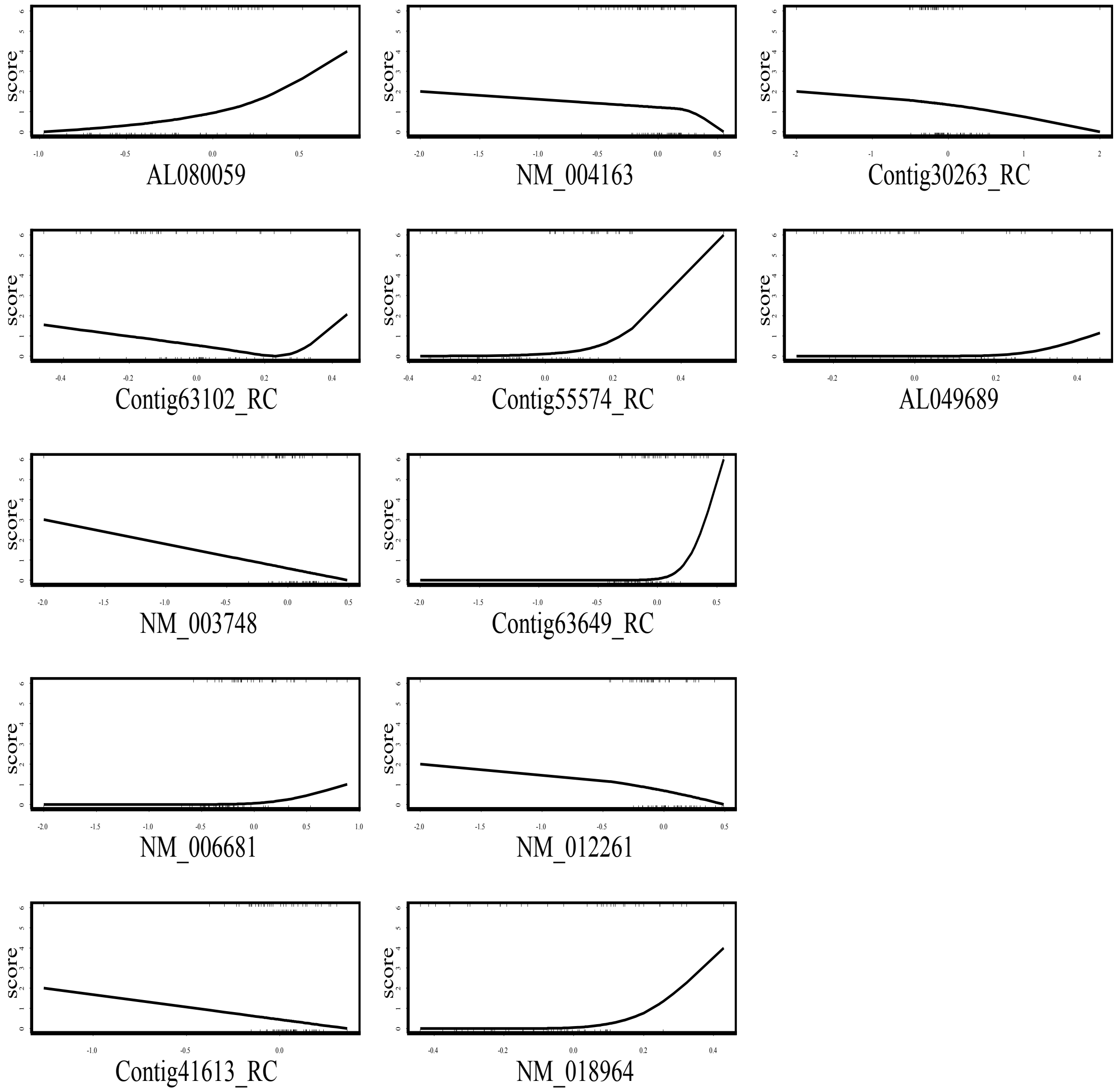

Figure 3A. Score plots of the 12 genes generated by pAUCBoost with $\overline{\alpha}_2 = 0.3$. The rug plot at the bottoms of each score plot shows observations of patients with good prognosis; the rug plot for patients with distant metastases is described at the top of each score plot.

Table 1A. *The genes selected based on* $P_g(100) > 0.5$ *for different values of* $\overline{\alpha}_2$ *with* $\overline{\alpha}_1$ *fixed to 0. The notation 1 indicates the gene is selected; 0 indicates the gene is not selected.*

No	gene name	$\overline{\alpha}_2 = 0.1$	$\overline{\alpha}_2 = 0.2$	$\overline{\alpha}_2 = 0.3$
1	Contig41613_RC	1	1	1
2	NM_006931	1	1	0
3	Contig40831_RC	1	1	0
4	Contig55574_RC	1	1	1
5	AB023173	1	1	0
6	Contig63649_RC	1	1	1
7	NM_018964	1	1	1
8	AL137615	1	0	0
9	NM_006201	1	0	0
10	NM_001710	1	0	0
11	AA555029_RC	1	1	0
12	Contig63102_RC	0	1	1
13	AL080059	0	1	1
14	NM_003748	0	1	1
15	NM_004163	0	1	1
16	Contig42421_RC	0	1	0
17	NM_000507	0	1	1
18	NM_012261	0	1	1
19	Contig37063_RC	0	1	1
20	NM_006681	0	1	1
21	AI355990_RC	0	0	1
22	Contig30263_RC	0	0	1
23	AL049689	0	0	1
